# From cortical and white matter structure to meaning: a brain-constrained neural network of semantic grounding in action and perception

**DOI:** 10.3389/fnhum.2026.1854823

**Published:** 2026-07-22

**Authors:** Rosario Tomasello, Ada D. Rezaki, Maxime Carriere, Lucius S. Fekonja, Friedemann Pulvermüller

**Affiliations:** 1 Brain Language Laboratory, Department of Philosophy and Humanities, WE4 Freie Universität Berlin, Berlin, Germany; 2 Cluster of Excellence: “Matters of Activity. Image Space Material”, Humboldt University, Berlin, Germany; 3 Cognitive Sciences, Department of Psychology, University of Potsdam, Potsdam, Germany; 4 Berlin School of Mind and Brain, Humboldt Universität zu Berlin, Berlin, Germany; 5 Department of Neurosurgery, Charité – University Hospital, Berlin, Germany; 6 Einstein Center for Neurosciences, Berlin, Germany

**Keywords:** brain-constrained neural networks, distributed neural assemblies, Hebbian learning, semantic grounding, sensorimotor regions, white matter, tractography-derived structural connectivity

## Abstract

The brain basis of semantic and conceptual processing is complex and difficult to explain. Specific word categories selectively engage some cortical areas, whereas others function as semantic hubs processing words across all categories. Beyond general neurobiological principles, cortical areas and their connectivity via white matter tracts are essential for determining an area’s role in language and semantic processing. Yet, most neural network models fall short of capturing the brain’s anatomical architecture, limiting their ability to provide mechanistic explanations. Here, we present a brain-constrained neural network model of 12 frontotemporal and occipital cortices constrained by tractography-derived structural connectivity, extending previous modelling work based primarily on literature-derived connectivity. Semantic circuits emerged spontaneously across the modelled cortical regions by means of Hebbian correlation learning, exhibiting distinct topographies: action words engaged fronto-central motor regions, while object words preferentially involved the primary visual area, replicating a range of neural activation patterns from neuroimaging studies. Crucially, regions central in the neural architecture, the anterior temporal and inferior prefrontal cortices, showed category-general semantic processing, consistent with a semantic hub function. A novel prediction concerns the potential hub-like contribution of the secondary temporo-occipital region, although this effect showed variability across tractography-derived structural connectivity variants. Correlation analyses further revealed that regions with richer inter-areal connectivity developed higher neural matter densities of the semantic circuits. Taken together, these findings demonstrate that brain-constrained neural models with increased biological realism at the white matter level can provide a mechanistic account of how distributed semantic representations emerge across multimodal hub, sensorimotor, and language regions.

## Introduction

Understanding a word such as *dog* requires linking a lexical form to its real-world referent and to a set of distinguishing semantic features (e.g., barks, has four legs, has a tail). Similarly, action-related words such as *grasp* may derive their meaning from the hand action to which they refer, including the characteristic motor patterns involved in grasping and manipulating objects. Other categories, including colour words (red), sound-related words (whisper), or emotion words (fear), may likewise be grounded in perceptual, motor, or affective experiences. From this grounding perspective, linguistic symbols are meaningful because they are intrinsically tied to sensorimotor experiences, making word meanings difficult to explain without reference to such experiential representations (Barsalou, 1999; Gallese and Lakoff, 2005; Harnad, 1990). By contrast, alternative accounts, the so-called amodal symbolic theories, argue that meaning is represented in abstract, modality-independent codes, whose computational efficiency derives precisely from being detached from perceptual and motor systems (Fodor, 1983; Machery, 2016; Mahon and Caramazza, 2008). On this account, the semantic content of words such as dog, grasp, or red is encoded without requiring grounding in sensorimotor experience, which has been argued to provide computational advantages. This theoretical debate has shaped much of the research in neurosemantics, with the central question being whether word meaning is inherently grounded in perceptual and motor neural representations or instantiated by an independent, abstract symbolic module.

Since then, much research has focused on identifying the neural correlates of semantic processing in the human brain in an attempt to resolve the debate. Neuroimaging studies show that symbolic processing engages a distributed cortical network extending beyond classical perisylvian language areas (Broca, 1861; Wernicke, 1874), with modality-preferential visual, auditory, and motor regions exhibiting meaning-specific activation patterns for visually, auditory, and motor-related words, respectively (Barsalou et al., 2003; Buccino et al., 2005; Carota et al., 2012; Chao et al., 1999; Damasio et al., 1996; García et al., 2019; Hauk et al., 2004; Kemmerer et al., 2012; Kiefer et al., 2008; Miranda et al., 2022; Moseley et al., 2013; Shebani et al., 2022; Vukovic et al., 2017). Although some studies and theoretical accounts have questioned or failed to demonstrate consistent sensorimotor involvement in semantic processing (de Zubicaray et al., 2013; Mahon and Caramazza, 2008; Postle et al., 2008), converging evidence from lesion and patients studies shows that damage to these modality-preferential regions results in selective word comprehension deficits (Damasio et al., 1996; Dreyer et al., 2015; Gainotti, 2010; Moguilner et al., 2021; Neininger and Pulvermüller, 2003; Roberts et al., 2017; Trumpp et al., 2013; Warrington and Mccarthy, 1983). Beyond sensorimotor involvement, research also indicates the presence of category-general semantic regions, often referred to as semantic hubs, where different types of word meanings are processed in a similar manner. Although the anterior temporal lobe has long been proposed as an amodal semantic hub (Mion et al., 2010; Patterson et al., 2007; Ralph et al., 2017), converging evidence suggests that hub-like semantic functions are not restricted to this region, but also involve the anterior inferior parietal and posterior inferior frontal cortices (Binder et al., 2009; Binder and Desai, 2011; Bookheimer, 2002; Carota et al., 2017; Fernandino et al., 2016; Schomers and Pulvermüller, 2016; Tate et al., 2014). Taken together, these findings raise the question of *why* modality-specific regions respond selectively to certain word types, whereas hub regions support general semantic processing, and *which* biological mechanisms underlie these processes.

Computational neural models provide a powerful tool for simulating symbolic learning and processing in the human cortex and for examining the emergence of functional roles of cortical regions. Although previous models successfully captured language and semantic patterns observed in human cognition (Chen et al., 2017; Christiansen and Chater, 2001; Dell et al., 1999; Elman, 2005; Guenther et al., 2006; Jackson et al., 2021; Plaut, 2003; Plaut and Gonnerman, 2000; Ueno et al., 2011), most of these models remained relatively distant from the cortical structure and the white matter pathways at different levels. Some models did incorporate connectivity structure constraints into their architectures (Chen et al., 2017; Jackson et al., 2021; Ueno et al., 2011), yet they often relied on biologically implausible learning mechanisms (backpropagation, Braitenberg and Schüz, 1998; Mazzoni et al., 1991; Raugel et al., 2026; Reilly, 1999) or did not incorporate the range of multimodal hub regions documented in previous studies (see for a review, Binder and Desai, 2011; Pulvermüller, 2013). Towards a genuine neuromechanistic explanation of symbolic processing, several scholars have therefore emphasised the need to implement brain structural and physiological constraints into model architectures to directly link neural architecture to emergent cognitive phenomena (Braitenberg, 1978; Breakspear, 2017; Palm, 1990; Pulvermüller et al., 2021; Reilly, 1999; van Albada et al., 2021).

Over the past years, much effort has been devoted to developing biologically constrained neural networks (BCNs) to provide a neuromechanistic account of language processing by mimicking key features of structural and functional properties of 12 cortical regions, including sensorimotor systems and multimodal semantic hubs (Carriere et al., 2025a, 2025b; Constant et al., 2023; Dobler et al., 2024; Garagnani et al., 2009; Garagnani and Pulvermüller, 2016; Henningsen-Schomers et al., 2023; Tomasello et al., 2017, 2018, 2026; Wennekers, 2007). These brain-constrained neural models have simulated the acquisition of object- and action-related words grounded in action and perception systems through biologically plausible Hebbian learning principles (Braitenberg, 1978; Hebb, 1949). Specifically, object and action words were learned through the co-activation of motor and visual cortical neurons, thereby mimicking real-world situations in which word forms become semantically linked with information about their corresponding objects and actions (Tomasello, 2003; Vouloumanos and Werker, 2009). As a result, these models exhibited the spontaneous formation of neural circuits, or cell assemblies (CAs, Palm et al., 2014) with distinct topographies: object-related CAs extended predominantly toward temporo-visual cortices, whereas action-related CAs were more prominent in frontal-motor regions, yielding category-specific activation patterns. Semantic hub regions, located centrally in the network architecture and characterised by a richer in- and outgoing connections (e.g., van den Heuvel and Sporns, 2013), exhibited higher neural CA cell density than secondary and primary regions, with moderate semantic category specificity. However, this specificity was less pronounced than in primary sensorimotor areas, indicating their role in general semantic processing across word types (Carriere et al., 2025b; Constant et al., 2023; Garagnani and Pulvermüller, 2016; Tomasello et al., 2017, 2018). Recently, these findings have also been replicated across different learning scenarios involving pre-exposure to word form and referents (Constant et al., 2023; see for discussion Tomasello, 2025) as well as in a NEST-based implementation (NEural Simulation Tool) of the same 12-area BCN model (Carriere et al., 2026). These BCN simulations demonstrate how two fundamental biological principles, white matter connectivity and Hebbian learning, give rise to category-specific semantic representations and category-general semantic hubs.

White matter (WM) tracts are widely considered essential for communication between local and distant cortical regions and for the emergence of higher cognitive functions in distributed neural networks (Catani et al., 2005; Catani and Bambini, 2014; Forkel et al., 2022, 2025; Forkel and Hagoort, 2024; Thiebaut de Schotten and Forkel, 2022). The relevance of human-specific WM tracts for language processing was demonstrated in simulations comparing a six-area perisylvian model with only next-neighbour connections to a more human-like architecture incorporating long-distance “jumping links” (Schomers et al., 2017). Guided by evidence for richer frontotemporal WM pathways in humans compared to non-human (e.g., chimpanzees) brains (Chauvel et al., 2026; Rilling, 2014; Rilling and Van Den, 2018), the human-like model produced larger cell assemblies, greater parallel activation, and sustained reactivation, a neural correlate of verbal working memory. These findings were later confirmed in larger spiking-network models of semantic learning (Carriere et al., 2025b). Similarly, the importance of brain-like connectivity became evident in the transition from mean-field models (Tomasello et al., 2017) to spiking neural networks (Tomasello et al., 2018), where additional cross-regional connections (jumping links) among 12 cortical areas were required to achieve functional symbolic processing. Converging evidence further highlights the role of rich white matter connectivity in hub regions, relative to primary and secondary areas, in enabling the rapid linking of word forms with referential information (Constant et al., 2023; see for a review, Tomasello, 2025). Together, these studies suggest that increased realism in connectivity structure enhances the biological plausibility, functional dynamics, and emergent topographies of neural circuit formation during learning.

However, the connectivity architecture of these previous models was based on a literature-based review of diffusion MRI studies in humans and non-human primates (see Table 1 in Tomasello et al., 2018 for an overview of these studies), which may have missed critical WM tracts relevant for language and semantic processing. Recent advances in tractography and whole-brain connectomics now provide more precise, empirically grounded maps of structural connectivity (Fan et al., 2016; Fekonja et al., 2019; Forkel et al., 2025). Although tractography has well-known limitations in fully reconstructing the human connectome, such as uncertainties in fibre orientation estimation, difficulties resolving crossing fibres, biases related to streamline length, and reduced sensitivity for thin or complex pathways (see, e.g., Dell’Acqua et al., 2025; Maier-Hein et al., 2017), it nevertheless offers valuable approximations of cortico-cortical and subcortical connectivity at the whole-brain level. One such resource is the Human Brainnetome Atlas, which provides a connectivity-based parcellation of the entire brain (Fan et al., 2016). Using probabilistic tractography applied to diffusion MRI data, the cortex was subdivided into 210 regions and the subcortex into 36 subregions. For each region, both functional and anatomical connectivity profiles were systematically characterised. Anatomical connectivity is represented as a binarised matrix (connection present vs. absent) derived from multi-subject diffusion tractography. A connection between two regions was defined as present if it was observed in more than 50% of participants. Otherwise, it was coded as absent. While this representation does not incorporate quantitative measures such as streamline counts or connection strength, it establishes a structural constraint by identifying which cortico-cortical pathways are consistently present across individuals. Integrating connectomic information of this kind enables neural models to move beyond the coarse, literature-based connectivity assumptions adopted in previous BCN modelling architectures, thereby increasing the neurobiological plausibility stepwise.

To this end, we extended a previous brain-constrained spiking neural network model, here called the “jumping links BCN model” (Tomasello et al., 2018, Figure 1A), mimicking 12 cortical areas in the left hemisphere across frontotemporal and occipital regions to simulate basic word acquisition of object and action words grounded in action and perception systems. Building on this, the present study advances previous neural architectures by replacing the literature-derived connectivity assumptions with empirical structural connectivity estimates from the Brainnetome Atlas (Fan et al., 2016). This connectomic extension represents the central novelty of the present work. It allows us to test whether the category-specific and category-general semantic topographies previously observed in the jumping links model are preserved, modified, or new predictions emerge when the same learning principles and cortical regions operate under tractography-constrained white matter connectivity. In doing so, the model provides a more anatomically grounded account of how distributed semantic circuits may emerge during symbolic learning, while also testing the robustness of the previous jumping links BCN architecture under increased biological realism.

**Figure 1:**
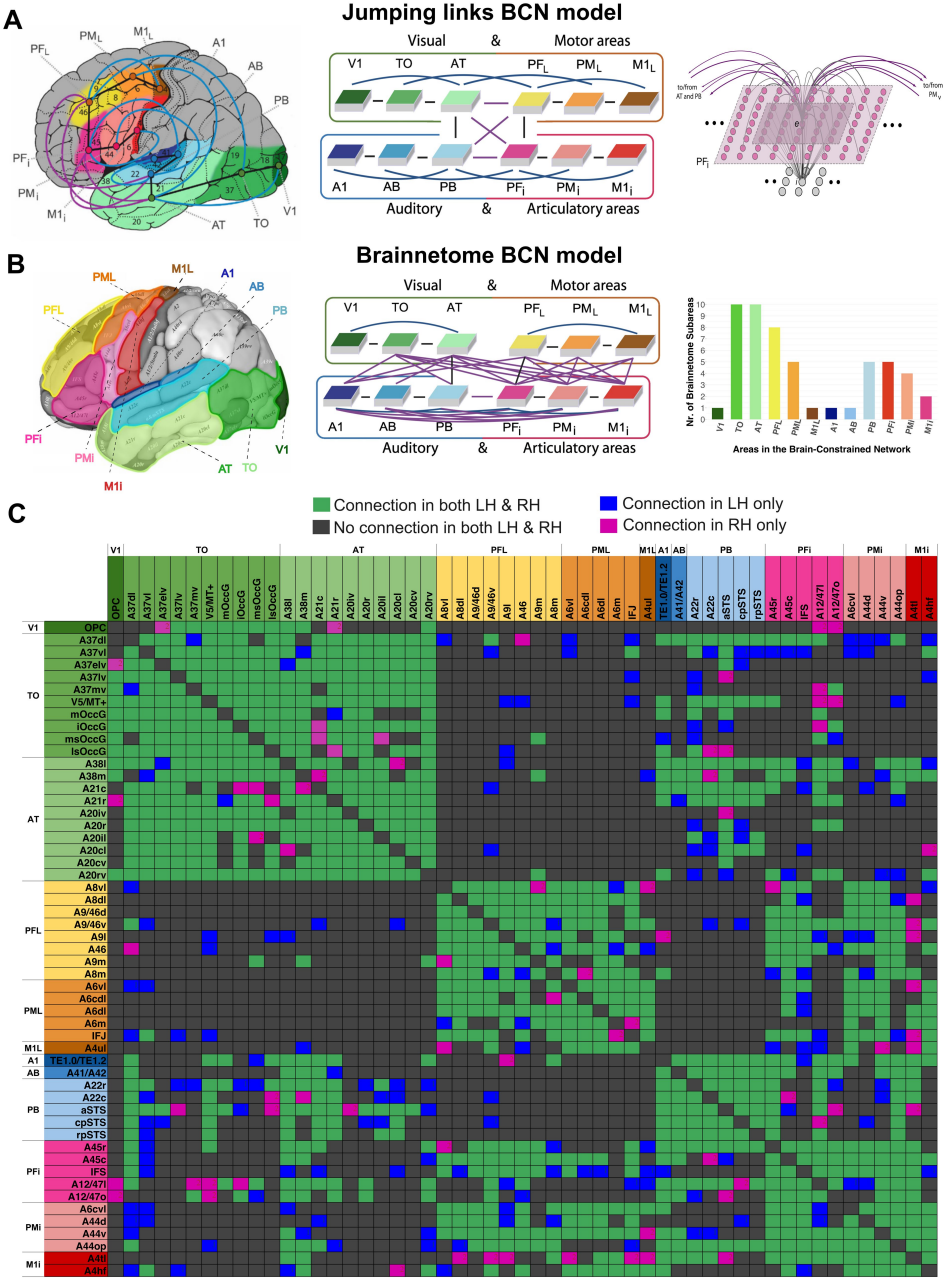
Cortical brain regions and connectivity structure in the Brain-Constrained Neural Network (BCN). **(A)** Twelve frontal, temporal, and occipital regions relevant for learning action- and object-related words are shown in the schematic brain (left). The perisylvian cortex comprises an articulatory–phonological system (red: M1i, PMi, PFi) and an acoustic–phonological system (blue: PB, AB, A1). The extrasylvian regions comprise a lateral motor system (yellow–brown: PFL, PML, M1L) and a ventral visual “what” stream (green: AT, TO, V1). Numbers indicate Brodmann areas (BAs), and arrows represent long-distance cortico-cortical connections identified in neuroanatomical studies and literature reviews. The middle panel shows the global area structure and connectivity of the “jumping links” model (Tomasello et al., 2018). Colours indicate correspondence between cortical and model regions. The right panel illustrates the micro-connectivity of one excitatory (e) neural element. Within-area excitatory links (grey) are restricted to a 19 × 19 local neighbourhood (light grey). Lateral inhibition is implemented by inhibitory cells (i), which suppress neighbouring excitatory elements in proportion to their local excitatory input (dark purple). **(B)** Mapping of the 12 modelled cortical regions onto the Brainnetome Atlas together with a schematic illustration of the model under increased tractography-based white matter connectivity constraints. The bar charts illustrate the number of Brainnetome subareas assigned to each BCN region, highlighting the finer granularity of the atlas compared to the 12-region model (with hub areas encompassing more subareas than primary and secondary regions). **(C)** The panel shows the left- and right-hemispheric connectivity matrices of the 53 Brainnetome subareas corresponding to the 12 BCN regions (Fan et al., 2016, full labels of the Brainnetome regions in Supplementary Table S1 in the Supplementary material). Green cells indicate connections present in both left and right hemispheres (LH & RH), blue cells indicate connections unique to LH, and magenta cells indicate connections unique to RH.

## Methods

### General features of the neural model architecture

The brain-constrained neural network (BCN) model was designed to approximate key structural and functional features of the human cortex at the micro (neural) and macro (system) levels, discussed to be relevant for simulating higher cognitive processes (Pulvermüller et al., 2021).

The general features included

1. **Model neurons:** Excitatory neurons incorporated mechanisms of spatio-temporal summation of their inputs, threshold-based spiking, and adaptation (Connors et al., 1982; Matthews, 2001).
2. **Hebbian synaptic modification:** Synaptic weights undergo changes based on Hebbian learning principles, including long-term potentiation (LTP) and long-term depression (LTD, Artola and Singer, 1993; Hebb, 1949).
3. **Inhibition and regulation:** Local and area-specific inhibitory mechanisms to regulate neural activity within and across areas (Braitenberg, 1978; Rubin et al., 2017; Yuille and Geiger, 2003).
4. **Within-area connectivity:** Sparse, random, and weak connections were present within each of the 12 areas. A neighbourhood bias favouring nearby connections was implemented (Braitenberg and Schüz, 1998; Kaas, 1997).
5. **Area structure:** 12 cortical areas relevant for language, conceptual, perceptual, and action-related processing in the human cortex were modelled.
6. **Between-area connectivity:** Connections between the 12 cortical regions were implemented based on the human Brainnetome Atlas (Fan et al., 2016, see Figure 1B), providing fine-grained anatomical information.
7. **Constant uniform uncorrelated noise:** All neurons in the network receive uniform uncorrelated noise constantly throughout learning and retrieval, as reported in biological systems (Rolls and Deco, 2010).

Detailed specifications of individual neuron properties, synaptic plasticity rules, and single-area architecture are provided in Appendix A in the Supplementary material and in a range of previous publications (Constant et al., 2023; Henningsen-Schomers and Pulvermüller, 2022; Tomasello et al., 2018, 2019, 2024, 2026). The parameters used can be found in Table S3 in the Supplementary material, and full implementation has recently been released as an open-access version within the NEST simulation framework (Carriere et al., 2026), facilitating transparency and reproducibility.

### Modelled cortical brain regions

The model comprised 12 cortical areas: six perisylvian language regions implicated in the acoustic and articulatory processing of spoken word forms (Fadiga et al., 2002; Pulvermüller, 1999; Pulvermüller and Fadiga, 2010; Zatorre et al., 1996) and six extrasylvian areas involved in visual object processing and motor action execution (Buccino et al., 2004; Deiber et al., 1991; Dum and Strick, 2005; Ungerleider and Haxby, 1994, see Figure 1A).

Perisylvian language system

- Auditory Superior Temporal areas: primary auditory cortex (A1, Broadmann area, BA41), secondary auditory belt (AB, BA42), and multimodal parabelt (PB, BA22).
- Articulatory Inferior Motor areas: inferior primary motor area (M1i, BA4), inferior premotor region (PMi, BA6/44), and multimodal inferior prefrontal hub (PFi, BA45).

Extrasylvian system

- Ventral Visual areas: primary visual cortex (V1, BA17), secondary temporo-occipital areas (TO, BA18/19/37), and multimodal anterior temporal areas (AT, BA20/21/38).
- Lateral Motor areas: lateral primary motor cortex (M1L, BA4), lateral premotor cortex (PML, BA6), and lateral prefrontal cortex (PFL, BA8/9/46).

Each of the 12 cortical areas in the model was composed of 625 excitatory pyramidal neurons (e-cells) and an equal number of inhibitory interneurons (i-cells), representing local pools of interneurons within the same cortical columns (Eggert and van Hemmen, 2000; Wilson and Cowan, 1972). Each cortical area consisted of a 25 × 25 grid of excitatory and inhibitory neurons, totalling 15,000 neurons across the entire network. Within each area, neurons were interconnected through sparse, random connections with neighbourhood bias, meaning spatially proximate neurons had higher connection probabilities, consistent with local connectivity patterns observed in the human cortex (Braitenberg and Schüz, 1998; Kaas, 1997).

### Connectivity structure

Previous BCN simulations using the 12-area semantic architecture, the “jumping links model” (Carriere et al., 2025b; Constant et al., 2023; Garagnani and Pulvermüller, 2016; Tomasello et al., 2018), examined semantic processing across different word types by implementing a connectivity structure between 12 modelled cortical areas. The connectivity was based on a range of neuroanatomical findings from small groups of humans and non-human primates using diffusion tensor and diffusion-weighted imaging (DTI/DWI) and deterministic tractography methods (see Table 1 in Tomasello et al. (2018) for an overview of these studies). The resulting connectivity structure incorporated next-neighbour area connections (black lines), “jumping links,” connecting second-next neighbour areas (blue lines), and long-distance corticocortical connections (purple lines, Figure 1A).

Recent advances in non-invasive neuroanatomy in large subject samples using diffusion MRI and probabilistic tractography enable comprehensive mapping of the brain’s structural connectome and offer opportunities to refine neurocomputational models with more precise anatomical constraints (e.g., van Albada et al., 2021). To this end, we extracted connectivity data from the Brainnetome Atlas (Fan et al., 2016), which parcellates the brain into 246 regions (210 cortical and 36 subcortical) and reports inter-area connectivity based on diffusion MRI tractography. In this atlas, a connection is defined as present if probabilistic tractography streamlines were consistently detected between two regions across participants, with the resulting matrices reported in both weighted and binary formats (1 = present, 0 = absent, for details of the calculation, see Fan et al., 2016). For the present simulations, we used the binary format and extracted inter-area connections corresponding to the left-hemispheric cortical regions of the 12-area semantic BCN model. Figure 1C compares binary connectivity patterns between the left and right hemispheres extracted from the Brainnetome Atlas. As a quality check, visual observation reveals that the left hemisphere generally exhibits richer inter-regional connectivity (blue cells) than the right hemisphere (magenta cells), consistent with well-established hemispheric asymmetries in the human brain (e.g., Hopkins and Rilling, 2000). Note that the present simulations were not designed to systematically test hemispheric lateralization. Therefore, this left-dominant connectivity pattern should be interpreted only as a descriptive observation based on the Brainnetome-derived connectivity data (Fan et al., 2016), rather than as an independent result of the present model.

The atlas of brain areas/nuclei and their connectivity was selected due to its close alignment with the model’s regional subdivisions. This potentially allows us to refine the BCN model’s connectivity structure compared to previous versions of the model. Note, however, that the alignment between the 12-area model and Brainnetome subdivisions was not always straightforward for some regions (see Figure 1B). For example, A1 and AB in the model were approximated by neighboring Brainnetome areas (TE1.0/TE1.2), PB was mapped onto a broader region (A41/A42) that also encompassed parts of AB, and M1i (A4tl, A4hf) was disproportionately large, overlapping substantially with the lateral motor subdivision (M1L), and thus not directly reflecting the typical motor strip subdivision (Fleming, 1938; Gordon et al., 2023; Rizzolatti, 2001). Such approximations inevitably introduce some uncertainty into the connectivity matrices. However, small variations within the atlas in assignments would affect only one or two links, thus not altering the overall connectivity pattern and results.

To extract relevant information from the Brainnetome Atlas and integrate anatomical connectivity data into the model, we followed a two-step process:

- **Identifying Regions of Interest:** We focused on the left hemisphere, comprising 105 cortical and 18 subcortical areas from the Brainnetome Atlas (see Figure S1 in Supplementary material). From this comprehensive dataset, 53 cortical subareas relevant to the modelled left frontotemporal and occipital cortices were identified through systematic visual inspection and anatomical correspondence mapping between the BCN model regions, their associated Brodmann areas, and the Brainnetome atlas parcellation scheme (Figures 1A,B and Table S1 in Supplementary material). These 53 subareas were then systematically grouped into 12 corresponding regions that mirror the BCN’s architecture, organised into primary, secondary, and multimodal hub regions (Figures 1B,C). The grouping revealed that multimodal hub areas encompassed significantly more Brainnetome subareas than primary and secondary regions (see bar plots in Figure 1B), reflecting their overall larger cortical size. Critically, we maintained strict non-overlapping assignments; no Brainnetome subarea was allocated to multiple BCN regions, ensuring clear regional boundaries.
- **Connectivity Matrices:** The structural connectivity information for the 53 selected subareas is represented as a binary matrix (Figure 1C), where 0 indicates no connection (dark grey), and 1 indicates a connection (green for both hemispheres, blue for left-hemisphere–only, and magenta for right-hemisphere–only), following the methodology of Fan et al. (2016). This 53 × 53 binary matrix served as the basis for constructing the connectivity structure of the 12 modelled regions. Specifically, connectivity data of all left hemisphere subareas (green and blue cells in Figure 1C) were grouped within each modelled region of the BCN and averaged to create aggregated connectivity profiles to accurately reflect each region’s connectivity pattern. For instance, if the AT (anterior temporal) area is represented by 10 subareas in the Brainnetome Atlas and 6 out of these 10 subareas are connected to the TO (temporo-occipital) region, then 6/10 = 0.6 (or 60%) was calculated as the connectivity percentage between AT and TO regions (see Figure S2 in Supplementary material). This procedure was applied to all possible pairs among the 12 BCN regions, yielding a mean connectivity matrix in which each cell represented the percentage of realised subregional connections between two cortical areas. However, because primary, secondary, and hub regions comprise different numbers of Brainnetome subregions, this approach may bias connectivity estimates: regions with more subregions have a higher probability of exhibiting at least one connection. For example, if only 1 out of 10 subregions within the anterior temporal lobe (AT) connects to another region, this single connection may not represent a robust or anatomically meaningful pathway at the level of the less fine-grained modelled cortical area. We therefore applied a thresholding criterion of 12.5%, such that a connection between two cortical regions was considered present only if at least 12.5% of all possible subregional pairs between them were structurally connected. If this proportion was not reached, the region-to-region connection was coded as absent (0).

For completeness, we also derived a binary no-threshold (fully dense) model by binarising the averaged connectivity matrix: a value of 0 indicated no connection, whereas 1 denoted the presence of at least one fibre tract between any pair of modelled regions. Specifically, if the mean connectivity between two regions exceeded 0%, that is, if at least one subregional connection was present, the corresponding cell was set to 1. In addition, we implemented a more conservative 25% threshold model, in which a region-to-region connection was defined as present only if at least 25% of all possible subregional pairs between the two regions were structurally linked (see Supplementary material, Figure S3).

Here, we focus on the Brainnetome-based 12.5% threshold model. This intermediate threshold was chosen to balance sensitivity and specificity: a no-threshold model risks including spurious or anatomically weak connections based on isolated subregional links, whereas the 25% threshold may be overly conservative and exclude biologically meaningful pathways that are reliably present but not sufficiently dense to surpass the higher cut-off. The 12.5% threshold was therefore used as a compromise that reduces the influence of isolated subregional links while preserving a broader set of plausible inter-areal connections. We do not interpret this value as an objectively optimal biological threshold, but rather as a principled aggregation criterion for mapping Brainnetome-derived connectivity onto the 12-area model. For completeness, and to assess the robustness of this choice, we also implemented no-threshold and 25% threshold variants. The simulation results and statistical analyses for these additional models are reported in the Supplementary material (see Figure S3).

#### Learning procedure

To simulate word acquisition in the Brainnetome-constrained neural network, we generated 12 independent neural networks. Each network represented a distinct simulated human cortex, with synaptic connections randomly initialized while remaining constrained by the Brainnetome-derived structural connectivity (12.5% threshold) and the local topographic neighbourhood principles described above. The number of simulated networks was based on previous BCN studies showing low variability across independently initialized models (Tomasello et al., 2017, 2018), similar to the present simulations.

To simulate word learning, sensorimotor patterns were implemented by activating 22 randomly selected neurons (3.5%) from the 625 excitatory cells in each primary cortical area, following the same learning procedure as in previous simulations (Carriere et al., 2025b, 2026; Garagnani and Pulvermüller, 2016; Tomasello et al., 2018). While the human brain is known to receive inputs that follow structured patterns, rather than being entirely random, the present study aimed to assess the potential for semantic learning of arbitrary symbol-referent mappings without imposing predefined input structures or assumptions. In this way, any semantic circuit topographies that emerged in the model arose from the interaction between Hebbian plasticity and the underlying connectivity architecture rather than from the way the input was structured. Each network instance employed 12 distinct sensorimotor pattern sets to model the learning of six action- and six object-related words. During learning, the primary perisylvian areas (the auditory (A1) and articulatory motor (M1i) regions) were simultaneously stimulated with one pattern each to mimic neural activity for spoken word processing: A1 stimulation captured acoustic-phonological word-form processing, while M1i stimulation modelled articulatory-phonological activity underlying speech production. Semantic grounding was implemented through concurrent activation of modality-specific areas: the primary visual cortex (V1) was stimulated during learning of object-related words, whereas the primary lateral motor cortex (M1L) was stimulated for action-related words, mimicking object perception and action execution, respectively, while a word is uttered. This learning setup reflects well-established learning scenarios in early word acquisition, in which words are spoken in the presence of corresponding objects or concurrently with related motor activities (Tomasello and Kruger, 1992; Vouloumanos and Werker, 2009). During each learning trial, three primary brain areas were directly activated by the input, while the fourth, modality-irrelevant area (M1L for object-related words and V1 for action-related words) received uncorrelated, variable noise input. This design reflects the neural variability of non-preferential modalities during word–referent learning. For example, when learning the word *“grasp,”* different objects may be grasped across experiences, leading to variability on the visual side while the motor component remains more consistently engaged. Accordingly, this design ensured a strong correlation between word-form activity in perisylvian language areas and referential information in the relevant modality (motor for action words; visual for object words), while keeping correlations with the non-relevant modality low. Such uncorrelated input patterns are critical, as it has been shown to prevent excessive neural circuit expansion into next-neighbourhood regions and support the emergence of distinct, topographically organised semantic representations (Doursat and Bienenstock, 2006; Tomasello et al., 2019, 2024).

Learning trials, in which a given word’s set of sensorimotor patterns was applied, were presented in random order. Training consisted of 12,000 learning trials, 1,000 presentations of each word’s patterns. Choice of this number was motivated by previous studies showing plateauing of learning above 1,000 trials (Garagnani et al., 2009; Nguyen et al., 2024; Schomers et al., 2017). During each learning trial, a word pattern was presented for 16 simulation time steps, followed by an interstimulus interval (ISI) during which no input was given. During the ISI, the network operated solely under general or unspecific noise (simulating spontaneous neural firing and/or inputs from other brain areas) and additional noise applied to the not-stimulated primary area. The next learning step was initiated only after global inhibition in the PFi and PB areas fell below a fixed threshold (0.65), allowing neural activity to return to baseline levels and preventing one trial from influencing the next. Global inhibition and the background spontaneous neural noise parameters were systematically increased to counteract the enhanced connectivity density across models related to the richer structural connectivity in the Brainnetome BCN model. The values were increased to 120 for global inhibition and 0.8 for background noise, relative to the previous model’s inhibition parameter of 60 and noise level of 0.5 (Tomasello et al., 2018). The increase in global inhibition (+100%; 60 → 120) was chosen to approximately match the near doubling of inter-area cortico-cortical connectivity (+91%; 22 → 42 unique links) introduced by the Brainnetome-based architecture. This proportional scaling was intended to preserve the excitation/inhibition (E/I) balance characteristic of cortical microcircuits (Braitenberg, 1978; Yuille and Geiger, 2003), ensuring that inhibitory activity increased in proportion to excitatory input. This prevented runaway excitation while maintaining sparse, distributed patterns of neural activity. Such parameter adjustments were necessary because the richer tractography-based connectivity otherwise produced excessive activation spread and unstable representations. Increasing inhibition and noise therefore ensured stable learning dynamics and reliable semantic circuit formation within the more densely connected Brainnetome architecture.

Nevertheless, to assess the impact of global inhibition and neural noise on neural circuit formation, we conducted parameter-control simulations for both the jumping links and Brainnetome models. In the jumping links model, five networks were simulated for each of four global inhibition levels (60–120, step size 20) and four background-noise levels (0–15, step size 5). The same procedure was applied to the Brainnetome model, with global inhibition ranging from 80 to 140 (step size 20) and background noise from 0 to 15 (step size 5; see Table S2 in the Supplementary material), resulting in 80 additional networks in total. These analyses were designed to determine whether changes in global inhibition and neural noise affected the spatial topography of the emerging cell assemblies (CAs), or primarily influenced their overall size and activation magnitude. Across both models, the results showed that global inhibition and noise mainly modulated CA size and overall activation strength after learning, while leaving the spatial topography of the emerging circuits largely unchanged (see Supplementary material, Figures S4, S5). This further supports the comparability of both models at the level of connectivity structure.

#### Cell assembly extraction and statistical analysis

During the acquisition of word meaning, strongly interconnected neuronal circuits known as cell assemblies (CAs) emerged across the modelled cortical areas through Hebbian synaptic plasticity. Following learning, these CAs were reactivated to simulate word production and to identify the neurons constituting each assembly. Reactivation was induced by simultaneously stimulating the learned word-form patterns in the primary auditory (A1) and articulatory motor (M1i) areas for two simulation time steps. During this retrieval phase, synaptic plasticity was disabled to prevent further weight modifications, ensuring that the observed activation patterns reflected the previously established cell assemblies rather than ongoing learning processes. No uncorrelated input pattern was given to the other primary regions (M1L for object and V1 for action) to capture specifically the cells belonging to a CA. Each extraction trial was followed by a 30-time-step inter-stimulus interval to allow the elicited activity to develop and then die out again.

For each modelled region, we determined the peak firing rate observed across all 625 excitatory cells within that region (in total, 7,500 e-cells). In estimating a cell’s average firing rate, we utilised the value w *_E_* (*e*,*t*) derived from Eq. (3.1 in Appendix A in the Supplementary material), incorporating a time constant $\tau_{\text{Favg}}$ = 15 (AVG_RATES_TIME and TAU_AVG_RATES). A specific excitatory cell (e-cell) was considered part of a given cell assembly (CA) circuit only if its time-averaged rate (referred to as “firing rate”) surpassed a threshold *θ* (with at least a peak firing rate of 0.2), which was both area- and input-pattern dependent. This threshold was defined as a fraction *γ* of the maximal time-averaged response of a single cell within that area to pattern *w*. More formally,

θ = θ_A_(w) = γ $\max_{x\in A}\bar{O{\left(x,t\right)}_{w}}$

where $\bar{O{\left(x,t\right)}_{w}}$ is the estimated time-averaged response of cell *x* to word pattern *w* (see Eq. 3.3 in Supplementary material Appendix A), and *γ* ∈ [0,1] is a constant (γ = 0.5 was used based on previous simulations, Garagnani et al., 2008, 2009; Tomasello et al., 2017, 2018). This was computed for each of the trained network instances and the estimated mean firing rate for each excitatory neuron in the model in response to each stimulation pattern. Cell assembly (CA) extraction followed previously established procedures (Garagnani et al., 2008; Henningsen-Schomers et al., 2023; Tomasello et al., 2018).

To statistically assess the CA size and topographical distribution across different regions, we averaged the six object-related and six action-related CAs separately. Differences between these mean CA sizes were examined using a four-way ANOVA with the following factors: WordType (2 levels: object, action) X PeriExtra (2 levels: perisylvian areas: A1, AB, PB, PFi, PMi, M1i, extrasylvian areas: V1, AT, TO, PFL, PML, M1L) X TempFront (2 levels: temporal areas: A1, AB, PB, TO, AT, V1, frontal areas: M1i, PMi, PFi, PFL, PML, M1L) X AreaType (3 levels: primary areas: A1, M1i, M1L, V1, secondary areas: AB, PMi, PML, TO, multimodal hub areas: PB, PFi, PFL, AT). Additionally, we performed further statistical analyses focused on the six perisylvian and six extrasylvian areas separately. For these analyses, we included the same factors: WordType, TempFront, and AreaType, excluding PeriExtra as a factor. To make the factorial structure of the ANOVA more transparent, Table 1 summarizes how each of the 12 model regions was assigned to the PeriExtra, TempFront, and AreaType factors.

**Table 1:** Mapping of the 12 model regions onto the WordType, PeriExtra, TempFront, and AreaType factors used in the four-way ANOVA.

Region	PeriExtra	TempFront	AreaType
A1—primary auditory cortex	Perisylvian	Temporal	Primary
AB—auditory belt	Perisylvian	Temporal	Secondary
PB—parabelt	Perisylvian	Temporal	Hub
M1i—inferior primary motor cortex	Perisylvian	Frontal	Primary
PMi—inferior premotor cortex	Perisylvian	Frontal	Secondary
PFi—inferior prefrontal cortex	Perisylvian	Frontal	Hub
V1—primary visual cortex	Extrasylvian	Temporal	Primary
TO—temporo-occipital cortex	Extrasylvian	Temporal	Secondary
AT—anterior temporal cortex	Extrasylvian	Temporal	Hub
M1L—lateral primary motor cortex	Extrasylvian	Frontal	Primary
PML—lateral premotor cortex	Extrasylvian	Frontal	Secondary
PFL—lateral prefrontal cortex	Extrasylvian	Frontal	Hub

WordType was defined by word category, with two levels: object and action. PeriExtra had two levels: perisylvian and extrasylvian; TempFront had two levels: temporal and frontal; and AreaType had three levels: primary, secondary, and hub.

Finally, to examine any relationship between the number of ingoing and outgoing connections of each modelled area on CA size, we conducted Pearson correlation analyses in RStudio using the stat_cor() function from the ggplot2 package (Wickham, 2016). The same correlation procedure was applied to the jumping links BCN model (Tomasello et al., 2018) to allow direct comparison across both neural architectures. In addition, the identical four-way ANOVA described above was conducted for the no-threshold and 25%-threshold Brainnetome BCN variants; these results are reported in the Supplementary material.

## Results

### Cell assembly size and topographies

**Figure 2:**
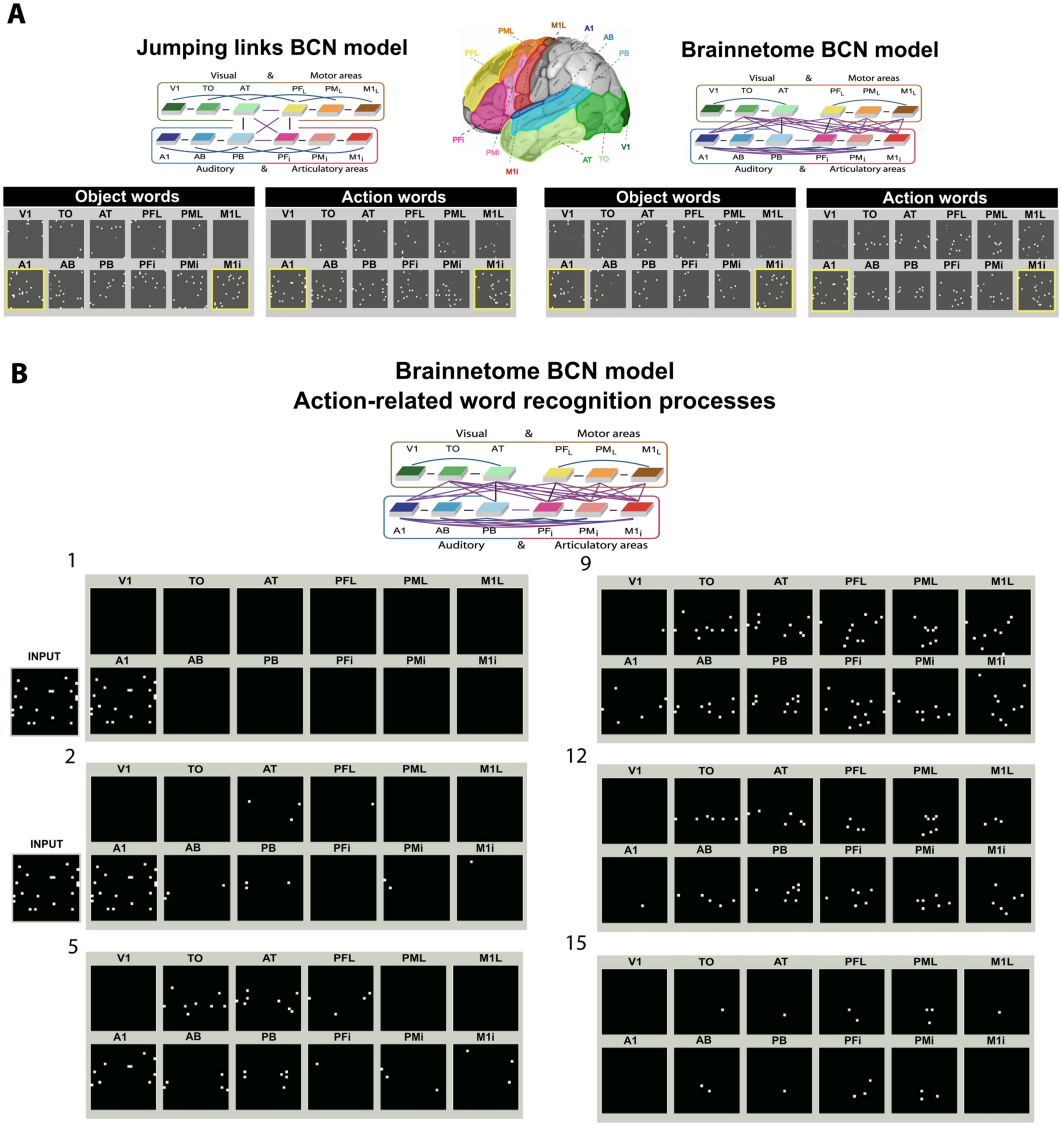
**(A)** Distribution of cell assemblies (CAs) emerging in the 12-area brain-constrained network (BCN) during word learning in the context of visual perception (object words) and action execution (action words). Left: jumping links BCN model; right: Brainnetome BCN model. Results are illustrated for one object word and one action word from a representative network instantiation for each model variant. Each set of 12 dark-grey squares corresponds to one modelled cortical area; white dots indicate the spatial distribution of CA neurons across regions. The top panel provides a schematic illustration of the jumping links and Brainnetome BCN architectures, alongside a cortical rendering highlighting the modelled regions. **(B)** Simulated auditory recognition for an action-related word (same example as in **A**). The network response was elicited by brief stimulation (2 time steps) of the learned auditory pattern in A1, resulting in full ignition of the distributed CA over time. As in **(A)**, the 12 cortical areas are represented as squares; here, activity is shown across selected time points. Each white pixel denotes a spike or a neuron belonging to the CA at a given time step.

**Figure 3:**
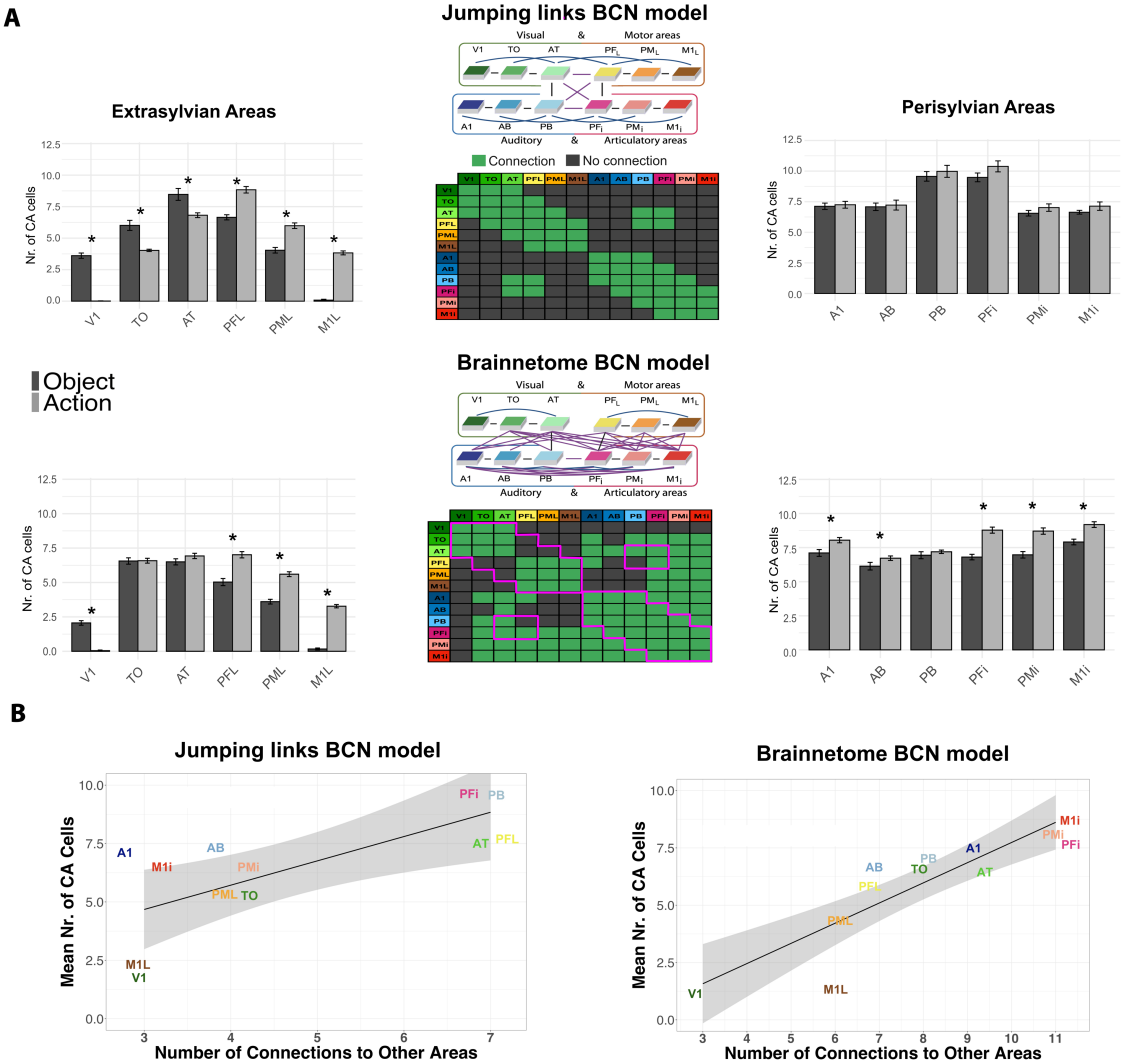
**(A)** Mean numbers of cell assembly neurons per area between the jumping links BCN model (Tomasello et al., 2018) and the Brainnetome BCN model, along with their connectivity matrices for direct comparisons. The jumping links model (top panel) features a connectivity structure derived from a literature review of neuroanatomical studies (see Table 1 in Tomasello et al., 2018), while the brainnetome model is based on whole-brain tractography data. In all connectivity matrices, green indicates the presence of connectivity between areas, while dark grey indicates the absence of connectivity. Magenta lines highlight the original connectivity structure of the jumping links model. Results for the extrasylvian areas are shown on the left and those for the perisylvian areas on the right. The bar plots show areas on the *x*-axis and the number of CA cells on the y-axis, with object words represented by dark grey bars and action words by light grey bars. Error bars indicate the standard error (SE). Asterisks indicate significant differences in the Bonferroni comparison of the number of CA neurons between object and action-related words. **(B)** Correlation between connectivity and cell assembly (CA) size. This figure shows the relationship between the number of incoming and outgoing connections (*x*-axis) and the mean number of CA cells (*y*-axis) for each of the modelled regions for both jumping links and Brainnetome neural models. Each scatterplot shows individual data points for 12 cortical areas, with regression lines indicating the direction and strength of the correlation between connectivity and CA size. The results show that regions with higher connectivity have a higher number of CA cells, while areas with fewer connections tend to have a smaller number of CA cells. *R* and *p*-values of the correlation analysis are reported.

The Brainnetome model architectures exhibited the spontaneous emergence of distributed cell assemblies (CAs) across multiple cortical regions by means of Hebbian learning, with each circuit selectively responding to specific sensorimotor input patterns (see Figures 2A,B). These emergent circuits linked word-form representations in perisylvian language cortices with semantic information in extrasylvian visual or lateral motor regions (see Figures 3A,B). In the following, we describe the results of the Brainnetome 12.5% model separately for the extrasylvian and perisylvian systems, focusing on CA cell density, that is, the number of cells within each region that were part of a given CA. The findings replicated from Tomasello et al. (2018), as well as the novel or differential findings observed across all Brainnetome models (12.5, 25%, and no-threshold; see Figure S3 in Supplementary material), are summarized in Table 2.

**Table 2:** Comparison of cell assembly (CA) topographies across modeled cortical regions between the previous jumping links and the Brainnetome models.

**Model type**	**Replicated cell assembly (CA) topographies**				**Novel findings**
	**Extrasylvian regions**		**Perisylvian regions**		
	**Visual**	**Motor**	**Auditory**	**Articulatory**	
**Jumping links model** *(literature-based connectivity; global inhibition = 60; noise = 5)*	**V1, TO:** Object > Action; **AT:** Moderate Object > Action;	**M1L, PML:** Action > Object; **PFL:** Moderate Action > Object;	**A1, AB, PB:** Object = Action;	**PFi, PMi, M1i:** Object = Action;	—
**Brainnetome 12.5% model** *(Brainnetome links ≥12.5%; global inhibition = 120; noise = 8)*	**V1:** Object > Action;	**M1L, PML:** Action > Object; **PFL:** Moderate Action > Object	**PB:** Object = Action;	—	**AT, TO:** Object = Action; **A1, AB, PFi, PMi, M1i:** Action > Objecs;
**Brainnetome no-threshold model** *(any non-zero Brainnetome link retained; global inhibition = 130; noise = 8)*	**V1:** Object > Action;	**M1L, PML:** Action > Object; **PFL:** Moderate Action > Object;	**A1, AB:** Object = Action;	—	**AT, TO:** Action > Object; **PB, PFi, PMi, M1i:** Action > Objecs;
**Brainnetome 25% model** *(Brainnetome links ≥25%; global inhibition = 120; noise = 8)*	**V1:** Object > Action;	**M1L, PML:** Action > Object; **PFL:** Moderate Action > Object;	**A1, AB, PB:** Object = Action;	—	**AT, TO:** Action > Object; **PFi, PMi, M1i:** Action > Objecs;

The table summarizes which CA topographies from the previous jumping links model of Tomasello et al. (2018) were replicated in the Brainnetome models and which findings differed or newly emerged under Brainnetome-derived connectivity. Connectivity assumptions and parameter settings are reported in the Model column. The two middle columns (extra- and perisylvian regions) list only those CA topographies that were replicated relative to the jumping links model; effects not listed for a given Brainnetome model were not replicated in that model. The final column lists novel or differential findings relative to Tomasello et al. (2018). “Object > Action” and “Action > Object” indicate higher CA cell density for one word type than the other, whereas “Object = Action” indicates comparable CA cell density for both word types. “Moderate” indicates smaller or less pronounced differences between word types.

**Extrasylvian regions:** The emergent CAs showed clear category-specific distributions, particularly in primary cortices: object-related words extended into primary visual cortex (V1) but not primary lateral motor cortex (M1L), whereas action-related words showed the opposite pattern, extending into M1L but not V1. Action words also recruited more neurons in the secondary motor region (PML), while the temporal–occipital region (TO) exhibited comparable CA cell densities for both word types (see Figure 3A, left lower panel).

The extrasylvian hub regions (AT and PFL) displayed higher overall CA cell densities than primary and secondary areas. The CA size distribution resembled that of the earlier jumping links model, although the characteristic “belly-shaped” pattern was less pronounced, with TO showing CA cell densities comparable to AT and PFL. PFL exhibited a slight preference for action-related words, whereas AT did not differentiate between word types, both of which primarly support category-general semantic processing.

**Perisylvian regions:** Within the perisylvian language system, action-related words produced higher CA cell densities in all articulatory motor regions (PFi, PMi, M1i), and in two auditory areas (A1 and PB). The two perisylvian hub regions (PFi and PB) did not show the same tendency toward larger numbers of CA cells, as observed in the jumping links model (see Figure 3A, right panels).

The different CA topographies were statistically investigated using a 4-way ANOVA (for more details, see Methods section) and were based on 11 networks, as one network was removed prior to statistical analysis due to merging of representations such that multiple CAs would coactivate and lead to excessive activation spreading, which rendered them impossible to analyse.

The 4-way ANOVA revealed a significant interaction between WordType, PeriExtra, FrontTemp, and AreaType (*F*(2, 20) = 39.75, *ε* = 0.64, *p* < 0.0001). Follow-up 3-way ANOVAs showed significant WordType × FrontTemp × AreaType interactions in both the extrasylvian (*F*(2, 20) = 39.73, ε = 0.59, *p* < 0.0001) and perisylvian systems (*F*(2, 20) = 10.60, ε = 0.91, *p* = 0.0007). A significant main effect of AreaType was found in the extrasylvian regions (*F*(2, 20) = 1056.58, ε = 0.91, *p* < 0.001), with hub regions showing the highest CA cell densities, followed by secondary and then primary regions (all *p* < 0.0001). A main effect of AreaType was also observed in the perisylvian regions (*F*(2, 20) = 30.41, ε = 0.74, *p* < 0.001), where primary areas exhibited higher CA cell densities than both secondary and hub regions (*p* < 0.0001), whereas secondary and hub regions did not differ significantly from one another (*p* = 0.07).

We further ran Bonferroni-corrected planned comparison tests (12 comparisons, corrected critical *p* < 0.0042) to further examine the differences in CA topographical distributions. This revealed significant differences in 4 of the 6 areas in the extrasylvian regions. In the visual system, primary V1 showed significantly higher CA cells for object words compared to action words (*p* < 0.0001), but this difference between word-related CA cells was not significant for the multimodal area AT and secondary TO region (*p* > 0.06). In the lateral motor system, we observed significant differences in all areas (M1L, PML, PFL), with action words CAs having significantly higher neural densities than object words (*p* < 0.0001). In the perisylvian system, action words formed significantly larger CA cells than object words in the articulatory motor regions (M1i, PMi, PFi; all *p* < 0.004). Action words also showed higher CA neuron densities in two auditory areas (A1, AB; *p* < 0.0009). No category difference was observed in the parabelt region (PB; *p* = 0.11, Figure 3A).

### Correlation results: connectivity richness and cell assembly size

To quantitatively assess the relationship between structural connectivity richness and area-specific neuron densities of the emergent cell assemblies, we conducted Pearson correlation analyses examining how the number of anatomical connections per simulated area predicts neuron densities of CAs in that area. These analyses were performed across all 12 modelled regions, and for both word types averaged together. Across all regions, the Brainnetome model revealed a strong positive correlation (r(10) = 0.89, *p* < 0.001), indicating a robust relationship between the number of cortico-cortical connections of a given area and the number of cell assembly (CA) neurons emerging within that region. For comparison, the same analysis was conducted for the jumping links BCN model (Tomasello et al., 2018, see Figure 3B), which similarly showed a significant correlation (r(10) = 0.90, *p* < 0.001). The magnitude of this association was comparable to that observed in the Brainnetome-constrained model, suggesting a consistent relationship between structural connectivity and the emerged CA size across architectures.

## Discussion

In the present study, we implemented a brain-constrained spiking neural network (BCN) comprising auditory, articulatory, visual, and motor cortical regions, whose cortico-cortical connectivity was constrained by tractography-derived data from the Human Brainnetome Atlas (Fan et al., 2016). The Brainnetome-informed BCN was used to simulate associative word learning, linking word forms to visually object or motor-related action information grounded in perception and action systems. Compared to a previous jumping links BCN model (Tomasello et al., 2018), which relied on a literature-based review of connectivity findings, the Brainnetome architecture exhibited an overall richer connectivity pattern both within and between auditory–articulatory perisylvian regions and visual–motor extrasylvian area (Figures 1A,B). However, several connections present in the earlier model were absent, specifically links between hub regions (AT and PFL) and secondary areas (TO and PML; see Figure 3A).

The brainnetome BCN model demonstrated the spontaneous formation of cell assemblies (CAs) with distinct topographies, connecting word-form representations in the perisylvian cortex with referential information in the visual and motor regions (Figures 2A,B). Specifically, object-related CAs extended predominantly into the primary visual area (V1) with little or no involvement of motor regions (PML, M1L). In contrast, action-related CAs extended more strongly into motor regions (PML, M1L) but not into V1, with the temporo-occipital region (TO) showing comparable activation between word types. Multimodal connector hubs (PFL and AT) exhibited higher CA densities than secondary regions, which in turn exceeded primary areas. AT showed no word-type differences, whereas PFL displayed a slight increase for action words. Because both hubs responded largely similarly to both word types, they functionally assumed the role of semantic hubs. A similar pattern emerged in TO, although this effect varied across the different Brainnetome model variants. Within the perisylvian system, action words preferentially engaged articulatory motor regions, as well as auditory A1 and PB areas. Finally, a significant positive correlation was observed between CA cell density and cortico-cortical connectivity richness across regions (see Figure 3B), indicating that structurally more connected areas developed a larger number of CA cells. The present tractography-constrained model replicates key findings from neuroimaging studies and previous BCN simulations, showing that category-specific and category-general semantic representations can emerge across varying degrees of biological realism. Below, we discuss these findings in relation to previous BCN architectures, empirical evidence, and methodological considerations.

### Tractography-based connectivity constraints in neural networks

Computational neural networks provide a powerful framework for investigating how large-scale neural populations give rise to language and semantic representations, offering insights into both processing mechanisms and associated impairments (Chen et al., 2017; Christiansen and Chater, 2001; Dell et al., 1999; Jackson et al., 2021; Rogers and McClelland, 2004; Ueno et al., 2011). Their explanatory power is particularly informative when network architectures more closely approximate the structural and functional properties of the human brain (Breakspear, 2017; Pezzulo et al., 2013; Pulvermüller et al., 2021; Tomasello, 2025; van Albada et al., 2021). In this regard, significant progress has been made with the development of brain-constrained neural models (BCN, Carriere et al., 2025a, 2025b, 2026; Constant et al., 2023; Dobler et al., 2024; Garagnani et al., 2008; Garagnani and Pulvermüller, 2016; Nguyen et al., 2024; Tomasello et al., 2017, 2018, 2026; Wennekers, 2007) that closely mimic the structural and functional properties of cortical regions involved in language and semantics (Figure 1A). Tomasello et al. (2018) simulated associative learning between spoken word forms and their object or action referents, mimicking basic word-learning scenario, in which an object is perceived or a manual action is performed while the corresponding word is spoken (Tomasello and Kruger, 1992; Vouloumanos and Werker, 2009). Object perception was implemented by stimulating the primary visual cortex (V1), whereas action execution involved stimulation of the primary lateral motor cortex (M1L), each paired with simultaneous activation of perisylvian auditory and articulatory regions (A1, M1i) to represent the spoken word form. This learning procedure yielded category-specific semantic circuits in perceptual and motor systems, alongside hub regions supporting category-general processing (for a review, Binder and Desai, 2011; Pulvermüller, 2013), highlighting the key role of connectivity structure and Hebbian learning in shaping semantic representations.

Here, we went one step further and enhanced the biological realism of the previous BCN by constraining cortico-cortical connectivity with tractography-derived data from the Brainnetome Atlas (Fan et al., 2016, Figure 1B). Relative to the earlier jumping links BCN architecture (Tomasello et al., 2018), the Brainnetome network exhibited markedly richer inter-regional connectivity (Figure 3), particularly between lateral motor and articulatory areas, as well as additional pathways linking auditory cortices. Despite the richer connectivity architecture, mimicking the same associative learning paradigm, pairing spoken word forms with object- or action-related perceptual and motor information, successfully led to the emergence of strongly interconnected cell assemblies (CA, Braitenberg, 1978; Garagnani et al., 2009; Hebb, 1949; Palm et al., 2014) grounded in the respective sensorimotor systems. Successful semantic circuits formation for both word types was demonstrated by the fact that short stimulation of only the auditory region (A1), mimicking auditory word recognition, was sufficient to reactivate the entire circuits, linking articulatory–acoustic word form representations with their semantic referential ones in primary motor or visual regions far away from input regions (see Figure 2B an example for action-related word; object words exhibit analogous neural activation dynamics).

At the same time, the richer tractography-based connectivity architecture required stronger inhibitory control and increased background noise to ensure stable circuit formation. Global inhibition was increased from 60 to 120 (100%) in the Brainnetome BCN model, approximately matching the near doubling of inter-area cortico-cortical connectivity introduced by the Brainnetome architecture (22 → 42 unique links, +91%), thereby preserving the excitation–inhibition balance of the previous jumping links model. Background noise was increased more conservatively (0.5 → 0.8), reflecting its role in decorrelating neural activity and preventing trivial fixed-point dynamics during learning and retrieval (Rolls and Deco, 2010). These adjustments reflect core neurobiological principles (Braitenberg, 1978; Rolls and Deco, 2010; Yuille and Geiger, 2003), according to which stable cortical processing depends on a balance between excitation, inhibition, and neural noise (Rubin et al., 2017). Without stronger inhibitory and noise regulation, the denser tractography-based connectivity produced excessive activation spread and merging representations, demonstrating that such mechanisms are essential for maintaining functional segregation and stable semantic circuit formation in richer and more biologically realistic connectivity architectures. All analyses were conducted within model types and between word categories, both of which were equally affected by these parameter adjustments.

Nevertheless, to directly assess whether changes in inhibition and neural noise could account for the observed the distinct CA distributions for object and actions words, we performed parameter-control simulations using the jumping links and the brainnetome models varying systematically global inhibition (GI) and background noise (N) (jumping links model, GI: 60, 80, 100, 120, and N: 0, 5, 10, 15; Brainnetome model, GI: 80, 100, 120, 140, and N: 0, 5, 10, 15, Table S2 in Supplementary material). The results showed that these parameter manipulations primarily influenced overall activation magnitude and CA size after learning in both model types, while the spatial distribution and category-specific topography of the emerging circuits remained comparatively stable (see Figures S4,5 in the Supplementary material). Notably, the Brainnetome model did not produce stable functional networks at lower inhibition values (GI = 80 and 100), supporting both the need for stronger inhibitory regulation in the denser architecture, and confirming the rationale for matching the increase in inhibition (+100%) to the increase in inter-area connectivity (+91%). This indicates that the CA topographies discussed in more depth below were not driven by the specific inhibition or noise values used in the simulations, but rather by the underlying connectivity architecture.

### Emergence of category-specificity and semantic hubs

A central finding of the present simulations is the spontaneous emergence of category-specific topographical activation patterns for both word types. Action-related cell assemblies (CA) extended preferentially into lateral motor cortices (PML, M1L) but not into primary visual cortex (V1), whereas object-related CA showed the contrary pattern, extending into V1 with minimal involvement of motor regions. This dissociation aligns with neuroimaging evidence demonstrating differential engagement of motor and visual systems for action versus object semantics, including contrasts such as tools versus animals (Binder and Desai, 2011; Garagnani et al., 2021; Klepp et al., 2019; Moseley et al., 2013; Pulvermüller, 2013; Pulvermüller and Fadiga, 2010; Shebani et al., 2022). The observed topographical differentiation depended on the uncorrelated input delivered to the non-preferential modality (V1 for action words; M1L for object words), which varied across learning trials. This variability approximates life-like word learning scenarios. For example, when learning a word such as “run,” the word form and the associated motor action remain consistently correlated across learning episodes, whereas the accompanying visual scenes and contexts may vary substantially from trial to trial. Consequently, Hebbian synaptic strengthening (long-term potentiation) occurred between correlated inputs from the relevant primary regions (Action: A1, M1i, M1L; Object: A1, M1i, V1), while uncorrelated inputs to V1 for action words and to M1L for object words promoted synaptic weakening (long-term depression). Hence, uncorrelated input acts as a regulatory constraint on circuit expansion, leading to category-specific semantic topographies (Doursat and Bienenstock, 2006; Tomasello et al., 2019, 2024). Conversely, when such uncorrelated visual input is absent, as in simulations modelling blindness (without visual input) during action-word learning, visual cortices become recruited for language-related processing (Tomasello et al., 2019, 2024), consistent with a range of fMRI findings (e.g., Amedi et al., 2003, 2004; Bedny et al., 2011).

We use the term “spontaneous emergence” not to imply that category-specificity arose independently of the learning contingencies, but rather that no predefined semantic topography or region-specific representational role was imposed on the network. Although Hebbian learning predicts that reliably co-activated patterns should become associated, several alternative outcomes were possible: learning could have remained largely local to the stimulated areas or to neighbouring regions; activation could have failed to propagate reliably across the network; different word-related assemblies could have merged or remained insufficiently differentiated, especially with richer connectivity architecture; or auditory word-form stimulation after learning might not have selectively reactivated the appropriate distributed semantic circuit. Thus, the observed CAs emerged from the interaction between correlation-based Hebbian plasticity and connectivity architecture, and are sufficient to form stable, category-differentiated semantic circuits grounded in sensorimotor experience. Note, however, that a large proportion of CA cells, that is, the number of neurons participating in a cell assembly within each region, were located in highly connected perisylvian language cortices and hub-like regions, whereas comparatively fewer CA cells were found in extrasylvian visual and motor cortices. This suggests that word meaning representations are more densely distributed within frontotemporal and multimodal hub regions. However, the present findings should not be interpreted as suggesting that semantic representations are localized exclusively in modality-specific sensory or motor cortices, nor that frontotemporal/perisylvian language regions operate independently from semantic processing. Rather, the model supports a distributed account of semantic representation spanning language-related, multimodal, and sensorimotor systems, rather than separate or independent semantic and language networks (Binder and Desai, 2011; Fernandino et al., 2016; Kuhnke et al., 2023; Pulvermüller, 2013; Ralph et al., 2017; Tomasello et al., 2017, 2018). In particular, the selective reactivation of V1 for object words and M1L for action words following word-form stimulation (Figures 3A,B) demonstrates that distinct word meanings become strongly differentiated within these modality-related regions, despite their comparatively lower CA cell densities.

Beyond category-specific sensorimotor effects, the extrasylvian regions AT and PFL exhibited a predominantly category-general semantic activation profile. In AT, both word types showed comparable CA densities, whereas PFL displayed only a moderate preference for action-related words over object-related ones. A similar pattern was observed in the earlier jumping links BCN model, which also showed attenuated category specificity in hub regions; however, AT exhibited a relative preference for object words (Carriere et al., 2025a, 2025b; Constant et al., 2023; Tomasello et al., 2017, 2018). Overall, these findings support the view that hub regions are involved in domain-general semantic processing rather than sharply differentiated category-specific representations. While the anterior temporal region (AT) has often been proposed as a central or unique semantic hub (Chen et al., 2017; Patterson et al., 2007; Ralph et al., 2017), the present results additionally account for the contribution of other hub-like regions, particularly PFL (see, e.g., Binder and Desai, 2011; Pulvermüller, 2013). A novel prediction of the present model is that the secondary TO visual area exhibits a comparable number of CA cells between word types, whereas the earlier jumping links model showed a relative preference for object-related words. In this respect, the model points to a potentially hub-like role for TO, possibly driven by its relatively rich connectivity profile, similar to that of established hub regions such as AT and PFL. However, this comparable number of CA cells in TO for action- and object-related words was not confirmed in the other two Brainnetome variants (no-threshold and 25% models), in which action-related CA cells were higher in TO and AT. Therefore, this finding should be interpreted cautiously. Nevertheless, it motivates future empirical investigations into the possible contribution of TO as a candidate semantic hub.

A further point of convergence with previous models concerns the overall distribution of CA cell densities across area types in the extrasylvian system. Similar to the jumping links BCN model, the present architecture exhibited a broadly “belly-shaped” profile, with the highest CA cell densities in hub regions, followed by secondary and then primary areas. Notably, TO and AT showed comparable CA cell densities, a pattern not observed previously. The elevated CA cell densities in these regions likely reflect both their integrative anatomical position, where auditory, articulatory, and visual or motor information converge, and their richer tractography-based connectivity, a defining feature of connector hubs supporting cognition (van den Heuvel and Sporns, 2013). This interpretation is supported by the correlation analysis: regions with more in- and outgoing connections showed higher CA cell densities, whereas sparsely connected areas showed lower values, regardless of connectivity constraints in neural architectures (Figure 3B). Thus, connectivity richness appears to promote the formation of larger cell assemblies, as converging inputs increase the likelihood of widespread co-activation and synaptic strengthening. However, other factors may also contribute and warrant further analysis. Anatomical location is one such factor, as centrally situated regions naturally receive converging inputs from multiple modalities, as discussed above. Network-level properties such as path length and the relative closeness of regions within the network may also play a role. Further, for example, the comparatively higher CA cell density observed in M1i among perisylvian regions may reflect both its richness in connectivity and its position as an input region.

Finally, although semantic differences were expected primarily within the extrasylvian system, category differences also emerged in perisylvian language cortices (auditory and articulatory regions). This contrasts with earlier simulations, which, due to their more symmetric connectivity, did not show such distinctions (Carriere et al., 2025b; Garagnani and Pulvermüller, 2016; Tomasello et al., 2017, 2018). In the Brainnetome-constrained model, action words consistently elicited higher CA cell densities in inferior motor cortices (PFi, PMi, M1i) and in two auditory regions (A1, AB), whereas PB showed no reliable word-type differences. These effects are best explained by the higher number of connections between inferior and lateral motor regions (motor–articulatory system) and the strengthened projections from auditory areas (A1, AB) to motor cortices. These findings suggest that action-related semantic representations may be more strongly embedded within classical perisylvian language cortices than previously assumed. However, these findings should be interpreted cautiously, as they may partly arise from the imperfect mapping between atlas-derived connectivity and the model’s cortical regions.

Specifically, some of the divergent outputs, most notably the overall stronger development of action-related CAs relative to the jumping links BCN model, may stem from the imperfect one-to-one mapping between some of the 12 modelled areas and Brainnetome atlas parcellation. Although the Brainnetome Atlas was chosen for its overall anatomical correspondence, several regions, particularly inferior and lateral primary motor areas (M1i, M1L), premotor cortex (PM), and auditory regions (A1/AB), required approximation when integrating tractography data (Figure 1B). For instance, A1 and AB were approximated by neighboring regions (TE1.0/TE1.2), while PB was mapped onto the broader A41/A42 region, which also encompassed parts of AB. Similarly, the subdivision between M1i and M1L was imperfect, as Brainnetome regions A4tl and A4hf (assigned to M1i) also extended into lateral M1L. This anatomical mixing may account for the richer connectivity observed among the six modelled motor cortices (articulatory and lateral motor regions), particularly the emergence of direct links between primary and secondary motor links (M1i↔M1L and PMi↔PML), which in turn likely favoured the stronger development of action-related CAs relative to object-related ones. This contrasts with the view that primary regions interact mainly via connector hubs or convergence zones in the human brain that integrate information across modalities (Sporns et al., 2007; van den Heuvel and Sporns, 2013). Here, the presence of direct motor–motor connections reduces reliance on hub-mediated integration and instead promotes direct communication.

Despite these limitations, the Brainnetome simulations replicated several key findings of earlier jumping links BCN models, including category-specific and category-general processing across cortical regions (Binder and Desai, 2011; Carota et al., 2024; Carriere et al., 2025b; Garagnani and Pulvermüller, 2016; Moseley et al., 2013; Pulvermüller, 2013; Tomasello et al., 2017, 2018). In particular, stronger emergence of action-related words in motor cortices (PFL, PML, and M1L) was consistently observed across all Brainnetome variants, representing the most robust finding across architectures and supporting a broad range of neuroimaging studies reporting motor system involvement in action semantics (see introduction). By contrast, greater variability emerged in visual regions, where the no-threshold and 25% variants showed relatively higher action-related CA cells in AT and TO compared with the 12.5% model (see Table 2 and Supplementary Figure S3). These differences likely reflect methodological trade-offs in constructing connectivity matrices and mapping atlas-based parcellations onto the modelled regions. For example, no-threshold approaches may introduce spurious subregional links, whereas stricter thresholds risk excluding anatomically meaningful pathways. In this respect, the 12.5% threshold model was selected as an intermediate aggregation criterion that may reduce isolated links while preserving a broad set of plausible inter-areal pathways, and it most closely replicated the results of the original jumping links model. Overall, the identification of biologically valid connections remains an important and widely discussed challenge in tractography-based connectomics (Dell’Acqua et al., 2025; Maier-Hein et al., 2017; Schilling et al., 2020, 2021), beyond the additional approximations involved in mapping atlas-based parcellations onto model cortical regions, as in the present work.

Beyond these issues, a tractography-based approach to neural modelling offers clear methodological advantages: it reduces researcher bias in selecting cortico-cortical connections and provides quantitative estimates of large-scale structural connectivity. With improved alignment between atlas parcellations and modelled regions, future implementations may achieve even closer correspondence between empirical connectomes and computational architectures. At the same time, although tractography-informed networks increase biological plausibility, they do not automatically enhance explanatory power. In some instances, more abstracted connectivity structures may yield results that are easier to interpret. Nevertheless, tractography-constrained BCNs open promising avenues specifically for constructing individualised “digital twins” by tailoring network architectures to single-subject connectivity data. Such personalised models hold potential for clinically relevant applications, including patient-specific prediction and intervention planning (Fekonja et al., 2024; Picht et al., 2021). First, however, modelling studies such as the present one are needed to determine whether tractography-derived connectivity can be meaningfully implemented in large-scale neural networks and still support stable and functional semantic circuit formation.

### Limitations and future perspectives

It is worth noting some limitations of the current modelling approach, as well as further possible extensions for future research. First, our model did not include additional cortical regions known to contribute to language and semantic processing, most notably the left inferior parietal cortex including supramarginal and angular gyri, which have been discussed both as additional semantic hub and as processing site for specific word categories, including prepositions, number words, and tool-related terms (Binder and Desai, 2011; Dehaene, 1995; Kemmerer et al., 2012; Shebani et al., 2017). However, adding the angular gyrus as an additional multimodal hub, alongside the anterior temporal lobe (AT) and posterior inferior frontal lobe (PFL), would likely yield similarly rich neural representations, characterised by high connectivity and dense cell assemblies reflecting its integrative role in combining visual, auditory, and motor information. Moreover, the connectivity structure was implemented as binary data, considering only the presence or absence of connections, without accounting for variations in connection strength or conduction delays, which have been documented in neurophysiological studies (Miller, 1994). Incorporating these finer connectivity dynamics and additional cortical regions could further enhance the model’s biological plausibility and provide a more comprehensive account of semantic representation. Additionally, future extensions could integrate sensorimotor input patterns derived from realistic objects, actions, and word form stimuli, enabling a more ecologically valid simulation of semantic learning and processing. Relatedly, the present simulations should be interpreted as a sufficient demonstration rather than as direct evidence that human word learning necessarily proceeds through tight, trial-by-trial, one-to-one correlated activity between perisylvian word-form areas and primary sensorimotor cortices. In real-life learning, such correlated activity is unlikely to be strictly one-to-one and may be substantially more variable. Thus, future simulations should examine how variations in the learning regime affect the formation, stability, and topography of semantic cell assemblies. Furthermore, the relatively larger number of CA cells in perisylvian language regions points to potential extensions of the model toward syntactic and combinatorial processing. If different words become linked into more complex linguistic structures, such mappings may preferentially emerge within perisylvian cortices because of their larger and more densely interconnected CA representations, consistent with the established role of these regions in syntactic and combinatorial language processing (Friederici, 2011; Moro et al., 2001; Schell et al., 2022). Despite its limitations, the present brain-constrained neural network replicated key neurocognitive findings on semantic processing and provides a biologically mechanistic account of how different cortical regions contribute to word meaning representation, further underscoring the explanatory value of brain-constrained neural networks for studying language and cognition.

## Conclusion

Previous modelling approaches proposed that the topographical distinction between action- and object-related semantic representations arises from the interplay of connectivity structure and Hebbian correlation learning (Garagnani and Pulvermüller, 2016; Pulvermüller, 2013; Tomasello et al., 2017, 2018). Previous simulations, however, relied on connectivity patterns derived from literature reviews. In the present study, we extended this line of work by modelling the same 12 frontotemporal and occipital cortices but constraining them with tractography-based connectivity data from the Brainnetome Atlas (Fan et al., 2016). The present model replicated core findings from prior modelling and neuroimaging research, including category-specific activations in sensorimotor cortices and the involvement of connector hubs in more general semantic processing. At the same time, it generated novel predictions: the TO region emerged as a potential additional connector hub alongside AT and PFL, and action-related representations were more strongly expressed in perisylvian regions than previously observed, both of which warrant further empirical examination. Overall, these findings underscore both the strengths and limitations of tractography-constrained BCNs. While such models reduce biases inherent in literature-based connectivity assumptions, they also inherit uncertainties related to atlas parcellations and their imperfect mapping onto modelled regions. Importantly, the present results help bridge brain anatomical structure, neurophysiological mechanisms, and linguistic-semantic theory in the study of word meaning, demonstrating how brain-constrained neural networks at varying levels of biological realism can inform mechanistic accounts of semantic representation.

## Data Availability

The datasets presented in this study can be found in online repositories. The names of the repository/repositories and accession number(s) can be found in the article/Supplementary material.
