# Supplementary material for: From cortical and white matter structure to meaning: a brain-constrained neural network of semantic grounding in action and perception
Source: Front Hum Neurosci. 2026 Jul 22;20:1854823. doi: 10.3389/fnhum.2026.1854823 (PMC13437771; doi:10.3389/fnhum.2026.1854823)
Supplement: Supplementary file 1 [file Data_Sheet_1.pdf]

From cortical and white matter structure to meaning: A brain-constrained neural network of semantic grounding in action and perception

Running title: Semantic processing in a brain-constrained neural network

Rosario Tomasello[1,2,3]*, Ada D. Rezaki[1,4], Maxime Carriere[1], Lucius S. Fekonja[2,5,] and Friedemann Pulvermüller[1,2,4,6]†

[1] Brain Language Laboratory, Department of Philosophy and Humanities, WE4 Freie Universität Berlin, Berlin, Germany
[2] Cluster of Excellence 'Matters of Activity. Image Space Material', Humboldt Universität zu Berlin, Berlin, Germany
[3] Cognitive Sciences, Department of Psychology, University of Potsdam, Potsdam, Germany
[4] Berlin School of Mind and Brain, Humboldt Universität zu Berlin, Berlin, Germany
[5] Department of Neurosurgery, Charité – University Hospital, Berlin, Germany
[6] Einstein Center for Neurosciences, Berlin, Germany

Corresponding author: *Rosario Tomasello rosario.tomasello@uni-potsdam.de

†Deceased 11.10.2025.

Data availability: Raw data of models' output and scripts are available at the following link: https://osf.io/zyk7t/overview

The full neural network model architecture is available as open-source software in:

Carrière, M., Dobler, F., Plesser, H. E., Feledyn, A., Tomasello, R., Wennekers, T., & Pulvermüller, F. (2026). A brain-constrained neural model of cognition and language with NEST: transitioning from the Felix framework. Cognitive Neurodynamics, 20, 48. and in https://github.com/MaximeCarriere/cogninest

Extrasylvian System

**Modelled regions**	**Abbreviation (Brainnetome atlas)**	**Full Name**
Primary visual area (V1)	OPC	Occipital pole cortex
secondary temporo-occipital (TO)	A37dl	Area 37, dorsolateral part
	A37vl	Area 37, ventrolateral part
	A37elv	Area 37, extreme lateroventral part
	A37mv	Area 37, medioventral part
	A37lv	Area 37, lateroventral part
	V5/MT+	Visual motion area V5/MT+
	mOccG	Middle occipital gyrus
	iOccG	Inferior occipital gyrus
	msOccG	Middle superior occipital gyrus
	lsOccG	Lateral superior occipital gyrus
anterior-temporal (AT)	A38l	Area 38, lateral part (temporal pole)
	A38m	Area 38, medial part (temporal pole)

	A21c	Area 21, caudal part
	A21r	Area 21, rostral part
	A20iv	Area 20, ventral intermediate subdivision
	A20r	Area 20, rostral part
	A20il	Area 20, lateral intermediate subdivision
	A20cl	Area 20, caudolateral part
	A20cv	Area 20, caudoventral part
	A20rv	Area 20, rostroventral part
lateral primary motor (M1L)	A4ul	Area 4, upper limb region
lateral premotor (PML)	A6vl	Area 6, ventrolateral part (premotor cortex)
	A6cdl	Area 6, caudal dorsolateral part
	A6dl	Area 6, dorsolateral part
	A6m	Area 6, medial part (SMA region)
	IFJ	Inferior frontal junction
lateral prefrontal (PFL)	A8vl	Area 8, ventrolateral part
	A8dl	Area 8, dorsolateral part
	A8m	Area 8, medial part
	A9/46d	Area 9/46, dorsal part (dorsolateral prefrontal cortex)
	A9/46v	Area 9/46, ventral part (dorsolateral prefrontal cortex)
	A46	Area 46 (middle frontal gyrus)

A9l	Area 9, lateral part
A9m	Area 9, medial part

Perisylvian Areas

**Modelled regions**	**Abbreviation (Brainnetome Atlas)**	**Full Region Name**
primary auditory (A1)	TE1.0	Primary auditory cortex, TE1.0 subdivision
	TE1.2	Primary auditory cortex, TE1.2 subdivision
Secondary auditory belt (AB)	A41	Area 41 (auditory cortex, Heschl's gyrus)
	A42	Area 42 (auditory association cortex, Heschl's gyrus)
parabelt (PB);	A22r	Area 22, rostral part (superior temporal gyrus)
	A22c	Area 22, caudal part (superior temporal gyrus)
	aSTS	Anterior superior temporal sulcus
	cpSTS	Caudal posterior superior temporal sulcus
	rpSTS	Rostral posterior superior temporal sulcus
inferior primary motor (M1i)	A4tl	Area 4, tongue and larynx region
	A4hf	Area 4, head and face region

inferior premotor (PMi)	A6cvl	Area 6, caudal ventrolateral part
	A44d	Area 44, dorsal part (pars opercularis, IFG)
	A44v	Area 44, ventral part (pars opercularis, IFG)
	A44op	Area 44, opercular part
inferior prefrontal (PFi)	A45r	Area 45, rostral part (pars triangularis, IFG)
	A45c	Area 45, caudal part (pars triangularis, IFG)
	IFS	Inferior frontal sulcus
	A12/47l	Area 12/47, lateral part (orbitofrontal cortex)
	A12/47o	Area 12/47, orbital part (orbitofrontal cortex)

Table S1. 53 cortical regions selected from the Brainnetome Atlas, grouped according to the modelled regions used in the present study. The table reports the corresponding Brainnetome labels and full anatomical names as provided in the original publication (Fan et al., 2016).

Left Intrahemispheric Connections in the Brainnetome Atlas

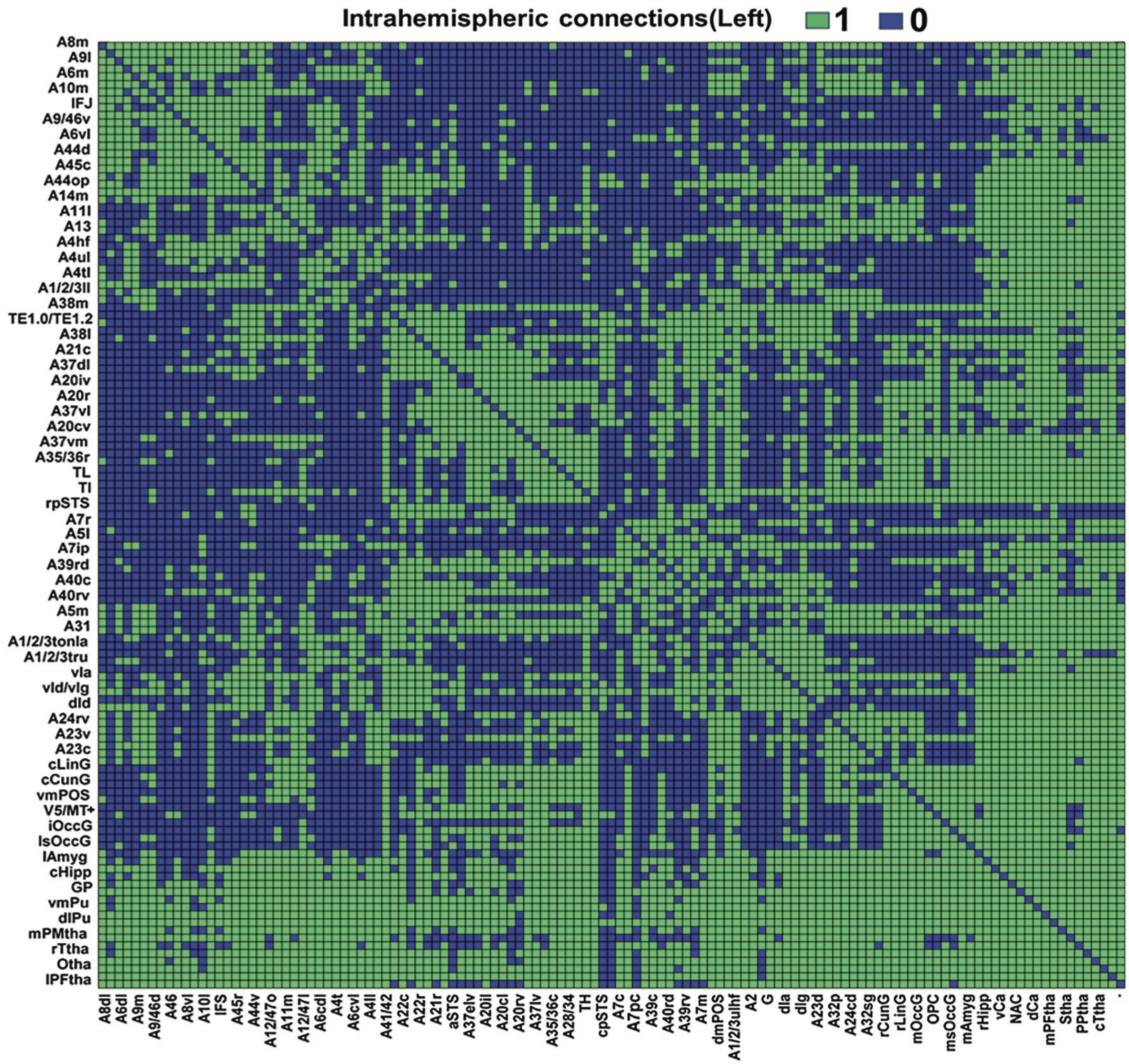

Figure S1. Left intra-hemispheric binary connectivity matrix of the 123 Brainnetome Atlas regions, adapted from Fan et al. (2016). Green indicates the presence of a connection, whereas blue indicates its absence.

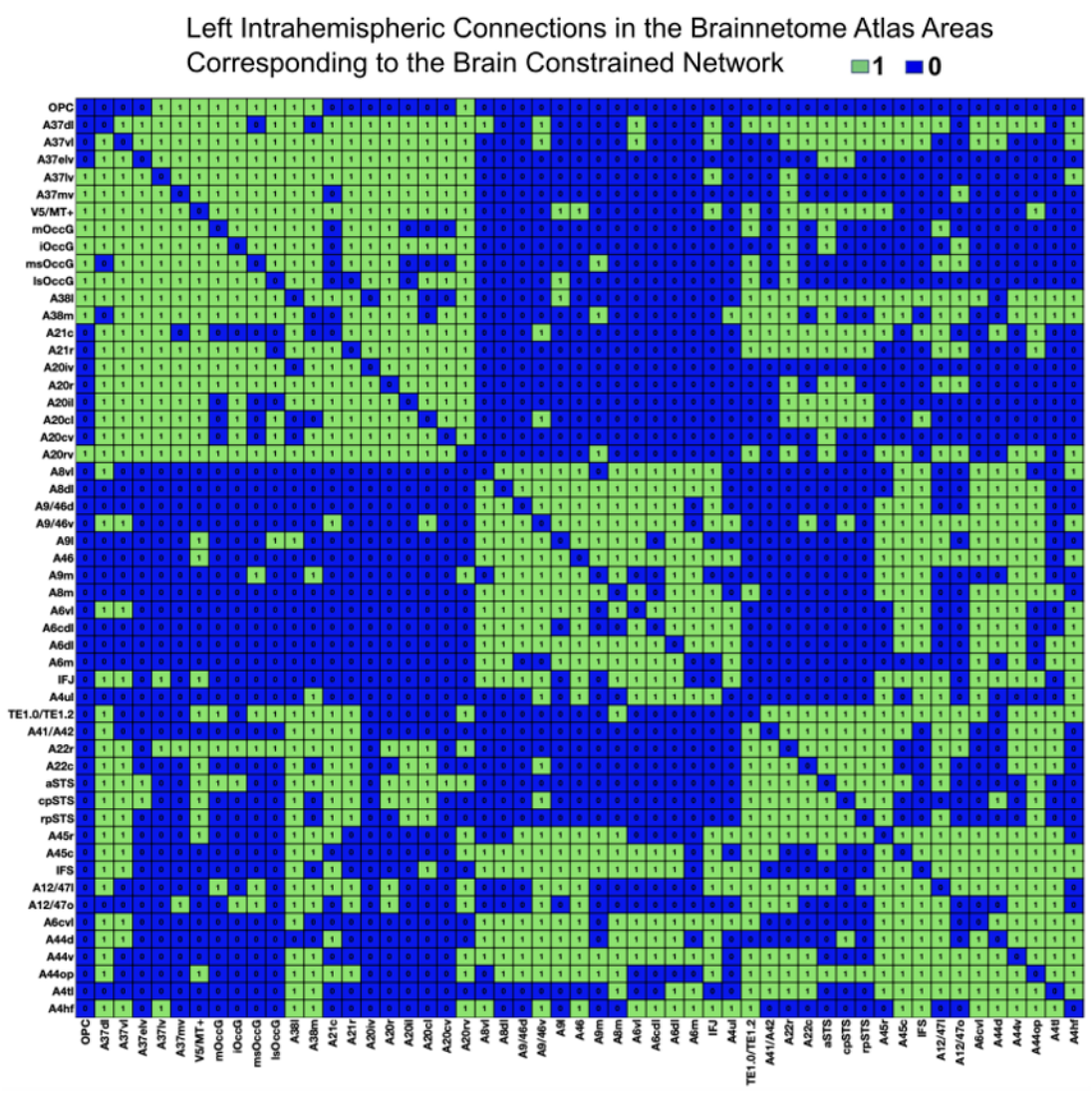

	V1	TO	AT	PFL	PML	M1L	A1	AB	PB	PFi	PMi	M1i
V1	0	0.7	0.3	0	0	0	0	0	0	0	0	0
TO	0.7	0.88	0.86	0.09	0.12	0	0.5	0.1	0.5	0.26	0.18	0.15
AT	0.3	0.86	0.8	0.06	0	0.1	0.5	0.4	0.66	0.42	0.25	0.25
PFL	0	0.09	0.06	0.84	0.8	0.25	0.13	0	0.05	0.68	0.91	0.25
PML	0	0.12	0	0.8	0.72	1	0	0	0	0.4	0.75	0.7
M1L	0	0	0.1	0.25	1	0	0	0	0	0.6	0.25	0.5
A1	0	0.5	0.5	0.125	0	0	0	1	1	1	0.75	1
AB	0	0.1	0.4	0	0	0	1	0	1	0.8	0.5	0.5
PB	0	0.5	0.66	0.05	0	0	1	1	0.8	0.44	0.4	0.2
PFi	0	0.26	0.42	0.68	0.4	0.6	1	0.8	0.44	0.8	0.9	0.7
PMi	0	0.18	0.25	0.91	0.75	0.25	0.75	0.5	0.4	0.9	0.75	1
M1i	0	0.15	0.25	0.25	0.7	0.5	1	0.5	0.2	0.7	1	0.5

	V1	TO	AT	PFL	PML	M1L	A1	AB	PB	PFi	PMi	M1i
V1	1	1	1	0	0	0	0	0	0	0	0	0
TO	1	1	1	1	1	0	1	1	1	1	1	1
AT	1	1	1	1	0	1	1	1	1	1	1	1
PFL	0	1	1	1	1	1	1	0	1	1	1	1
PML	0	1	0	1	1	1	0	0	0	1	1	1
M1L	0	0	1	1	1	1	0	0	0	1	1	1
A1	0	1	1	1	0	0	1	1	1	1	1	1
AB	0	1	1	0	0	0	1	1	1	1	1	1
PB	0	1	1	1	0	0	1	1	1	1	1	1
PFi	0	1	1	1	1	1	1	1	1	1	1	1
PMi	0	1	1	1	1	1	1	1	1	1	1	1
M1i	0	1	1	1	1	1	1	1	1	1	1	1

Figure S2. Left-hemispheric connectivity matrices for the 53 Brainnetome regions corresponding to the modelled regions (Left panel). Aggregated connectivity profiles obtained by averaging as described in the manuscript, for each modelled region (Upper right panel). The resulting binary "no-threshold" matrix between the 12 modelled regions (Lower right panel:), which served as the basis for constructing the two thresholded Brainnetome variants (12.5% and 25%).

Results of the no-threshold and 25% thresholded model

Two additional Brainnetome-informed BCN variants were implemented using no-threshold and 25%-threshold connectivity matrices. For each variant, 12 independent networks were instantiated to simulate basic word–referent mappings grounded in action and perception systems (see main text). To ensure stable network dynamics across architectures with different connection densities, global inhibition and background noise parameters were adjusted accordingly. The no-threshold model, characterised by the densest connectivity, required the strongest inhibition (130) combined with background noise of 0.8. In contrast, the 25%-threshold model operated with slightly lower inhibition (120) and the same noise level (0.8), as for the main 12.5% Brainnetome model. For comparison, the earlier jumping-links BCN model (Tomasello et al., 2018) used substantially lower inhibition (60) and noise (0.5).

Both variants showed successful learning, with the spontaneous emergence of distributed cell assemblies (CAs) linking word-form representations to their corresponding object or action referents in visual and motor cortices. Overall, these models exhibited CA distributions comparable to the main Brainnetome model in the extrasylvian system, particularly the higher CA densities for action-related words in motor regions (M1L, PML, PFL) relative to object-related words. Primary visual cortex (V1) consistently showed higher CA densities for object-related words across models. However, in the no-threshold and 25% threshold variants, TO and AT displayed slightly higher CA densities for action-related words, a pattern not observed in the 12.5% threshold model. In all cases, these word-type differences remained modest. In the perisylvian cortical regions, consistent with the 12.5% threshold model, higher CA cell densities for action words were observed in articulatory motor areas (PFi, PMi, M1i), whereas auditory regions showed variable patterns across word types (see Fig. S3).

These observations were confirmed by the statistical analysis described above.

No threshold model: The 4-way ANOVA revealed a significant interaction between WordType, PeriExtra, FrontTemp, and AreaType ($F(2,12) = 28.40$, $\varepsilon = 0.89$, $p < 0.001$), indicating differential CA topographies for action and object words across cortical areas. To examine these effects further, separate 3-way ANOVAs were conducted for peri- and extrasylvian systems. A significant WordType × FrontTemp × AreaType interaction emerged in the extrasylvian system ($F(2,12) = 100.79$, $\varepsilon = 0.96$, $p < 0.001$), but not in the perisylvian system ($F(2,12) = 0.71$, $\varepsilon = 0.96$, $p = 0.93$).

In the extrasylvian regions, a main effect of AreaType was observed ($F(2,12) = 63.86$, $\varepsilon = 0.77$, $p < 0.001$), with hub regions showing the highest CA densities, followed by secondary and then primary regions. In the perisylvian system, AreaType was also significant ($F(2,12) = 380.66$, $\varepsilon = 0.63$, $p < 0.001$), but here primary regions exhibited

higher CA densities than secondary and hub regions ($p<0.001$), which did not differ from each other ($p=1$).

Bonferroni-corrected planned comparisons (12 comparisons, corrected critical $p < 0.0042$) showed word-type effects across several regions. In the extrasylvian system, object words formed larger CAs in primary visual cortex (V1, $p < 0.0001$), whereas action words produced larger CAs in lateral motor (M1L, PML, PFL) and visual association areas (AT, TO, $p < 0.0001$). In the perisylvian system, planned comparisons revealed significant differences in the articulatory motor regions (M1i, PMi, PFi) and in the parabelt region (PB), where action words again showed significantly larger CAs (all $p < 0.0014$). By contrast, no differences were observed in the primary auditory cortex (A1) or the auditory belt (AB) (all $p > 0.0094$, Fig. S3, upper panel). Although the overall perisylvian interaction in the ANOVA was not significant, these comparisons are reported for completeness.

25% threshold model: Replicating the pattern observed in the previous models, the 4-way ANOVA again revealed a significant interaction of WordType, PeriExtra, FrontTemp, and AreaType ($F(2,22) = 23.16$, $\varepsilon = 0.95$, $p < 0.0001$). Follow-up 3-way ANOVAs showed a significant WordType × FrontTemp × AreaType interaction in the extrasylvian system ($F(2,22) = 57.17$, $\varepsilon = 0.83$, $p < 0.0001$), but not in the perisylvian system ($F(2,22) = 1.03$, $\varepsilon = 0.84$, $p = 0.37$). A robust main effect of AreaType was again observed in the extrasylvian regions ($F(2,22) = 1033.1$, $\varepsilon = 0.79$, $p < 0.0001$), with hubs showing the highest CA densities, followed by secondary and primary areas ($p < 0.0001$). In the perisylvian system, AreaType was also significant ($F(2,12) = 13.72$, $\varepsilon = 0.98$, $p < 0.001$), with hub regions exhibiting higher CA densities than primary and secondary regions ($p < 0.0001$), though no significant difference was found between primary and secondary regions ($p = 0.08$).

Bonferroni-corrected planned comparisons (12 comparisons; corrected $p < 0.0042$) revealed word-type differences across several regions. In the extrasylvian visual system, V1 showed larger CAs for object than action words ($p < 0.0001$), TO showed no difference ($p = 0.52$), and AT showed larger CAs for action words ($p = 0.0017$). In the extrasylvian lateral motor system, M1L, PML, and PFL all showed larger CAs for action words (all $p < 0.001$, Fig. S3, bottom panel). In the perisylvian system, significant word-type effects were confined to articulatory motor areas (M1i, PMi, PFi), where action words yielded larger CAs (all $p < 0.001$), while no differences were observed in auditory regions (A1, AB, PB; all $p > 0.01$). These comparisons are reported for completeness despite the non-significant overall ANOVA interaction in the perisylvian system.

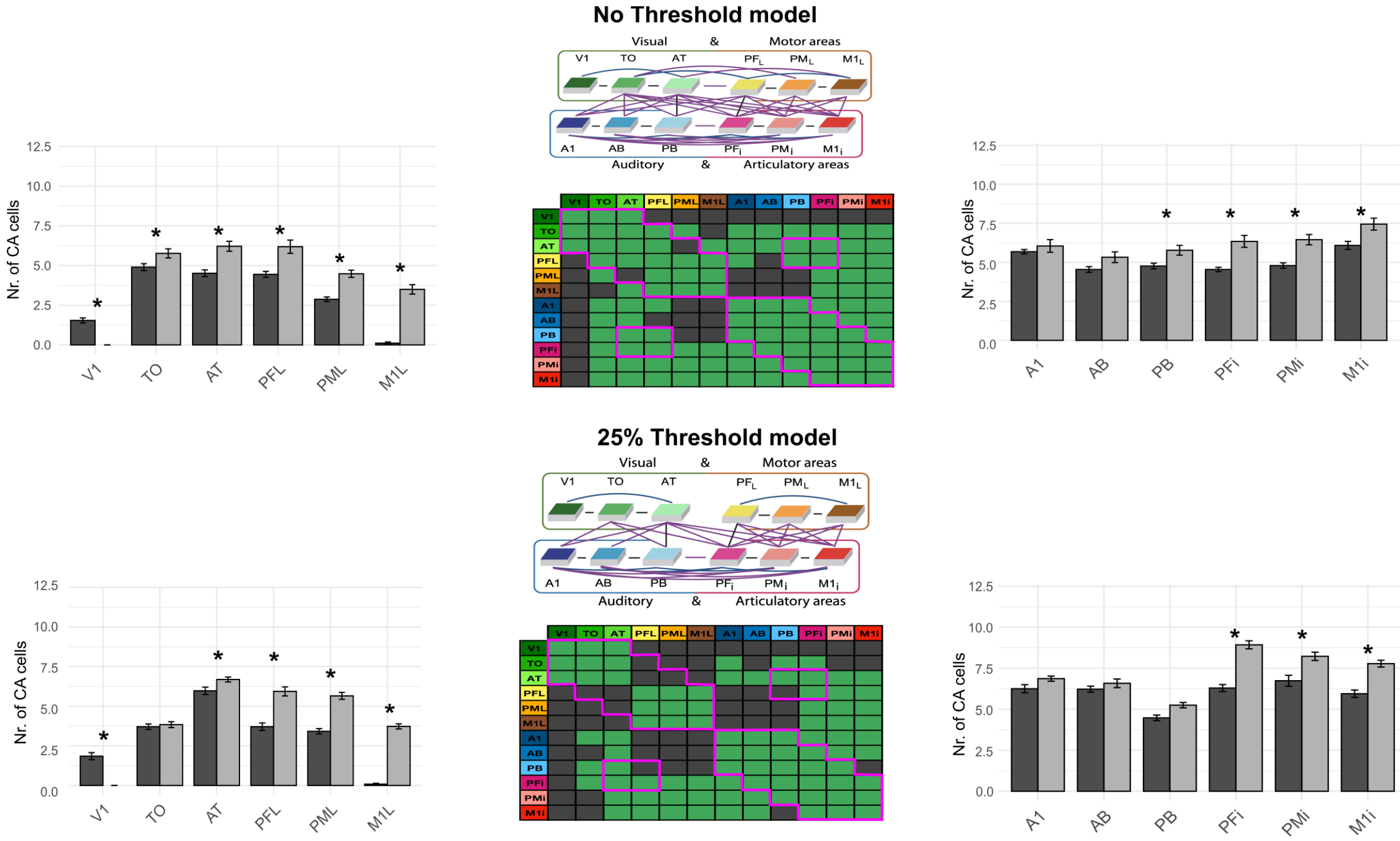

Figure S3: Distribution of cell assemblies (CAs) emerging in the 12-area network during word learning in the semantic context of visual perception (object words) and action execution (action words). Mean number of cell assembly neurons per area between the No-threshold and 25%-thresholded Brainnetome model, along with their connectivity matrices for direct comparisons. In all matrices, green indicates the presence of connectivity between areas, while dark grey indicates the absence of connectivity. Results for the extrasylvian areas are shown on the left and those for the perisylvian areas on the right. The bar plots show areas on the x-axis and the number of CA cells on the y-axis, with object words represented by dark grey bars and action words by light grey bars. Error bars indicate the standard error (SE). Asterisks indicate significant differences in the Bonferroni comparison of the number of CA neurons between object and action-related words.

Parameters Analysis of changes in Global Inhibition and Neural Noise

Model	Parameters	Functional Networks
Jumping links Model (Tomasello et al 2018)	Noise = 0	4
	Noise = 5	4
	Noise = 10	5
	Noise = 15	4
	GI = 60	4
	GI = 80	4
	GI = 100	4
	GI = 120	4
Brainnetome 12.5% Model	Noise = 0	4
	Noise = 5	5
	Noise = 10	4
	Noise = 15	5
	GI = 80	0 (merged activity)
	GI = 100	0 (merged activity)
	GI = 120	5
	GI = 140	5

Table S2. Number of functional networks obtained in the parameter-control simulations for the jumping links and Brainnetome models under different global inhibition (GI) and background noise conditions. All simulations initially started with five independently initialized networks per condition. Reduced numbers reflect networks excluded because of merged or unstable activity patterns, particularly at lower inhibition values in the Brainnetome architecture.

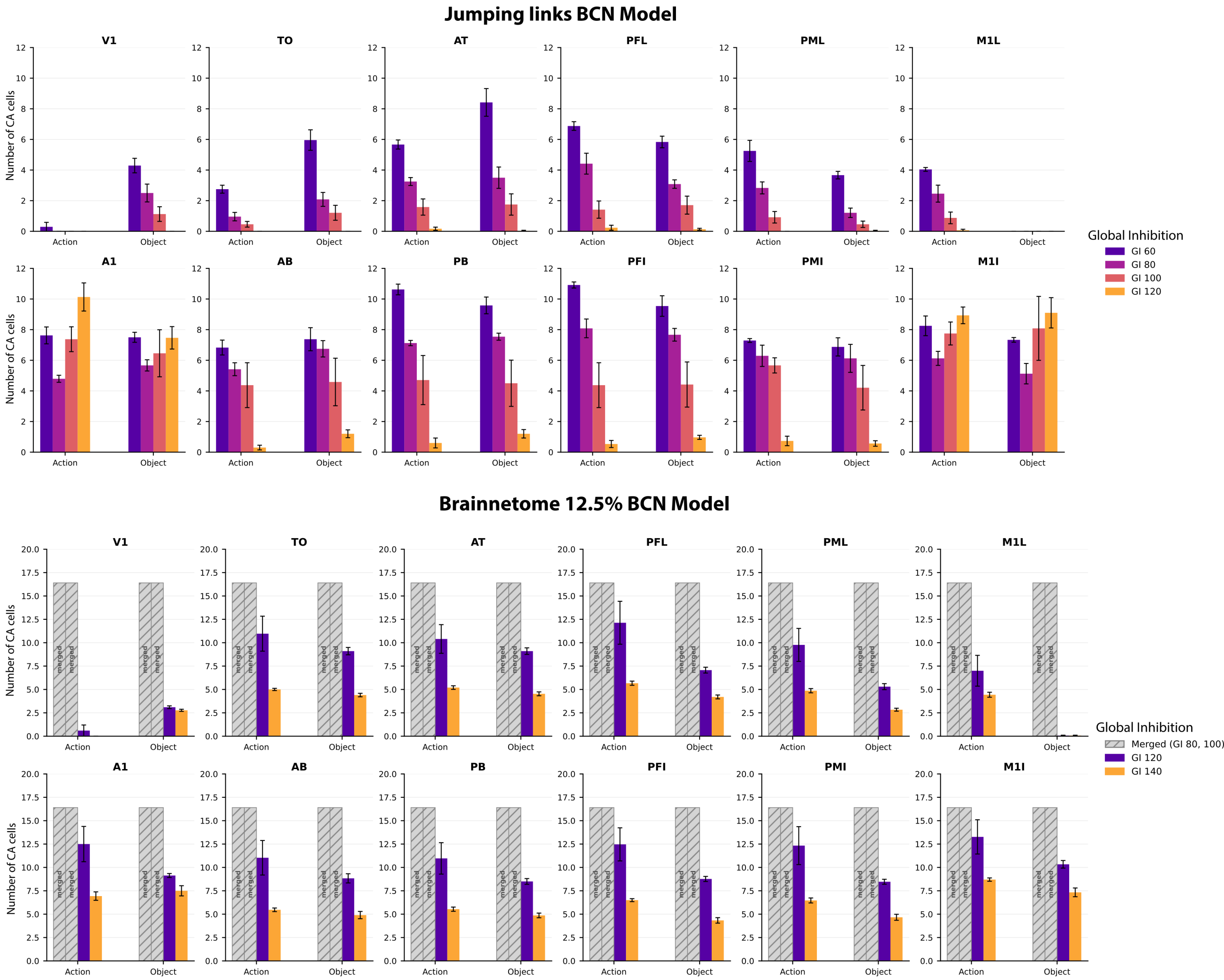

Figure S4. Impact of global inhibition in cell assembly formation. The figure illustrates the effects of varying global inhibition (GI) on the formation of cell assemblies (CAs) for action- and object-related words in the jumping links and brainnetome model. Global inhibition was varied from 60 to 140 in steps of 2, differently for the two model types (see Table S2). The results show that changes in global inhibition primarily affected overall CA size and activation magnitude, while having comparatively little impact on the topographical distribution differences between action- and object-related word representations.

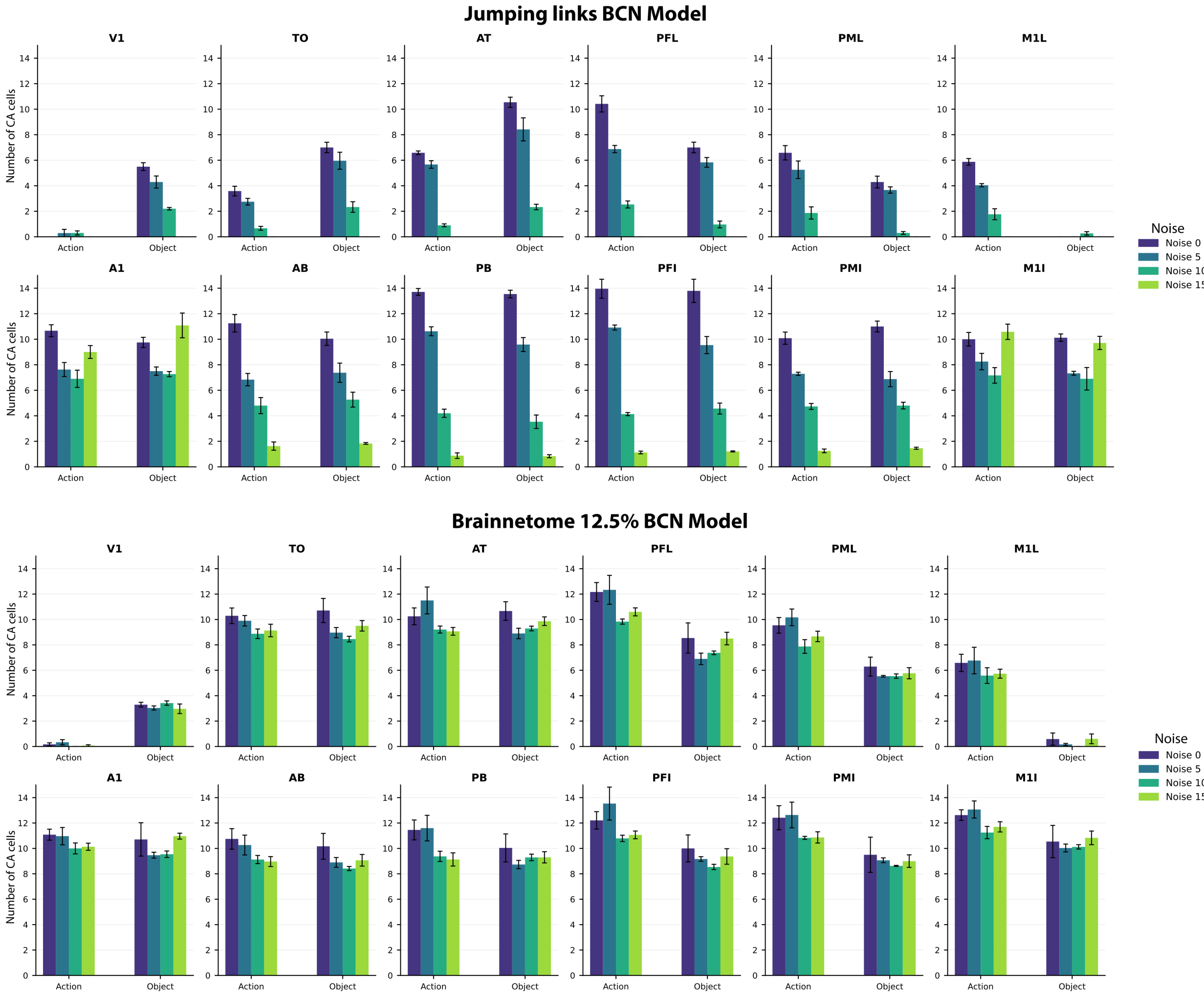

Figure S5. Impact of neural background noise in cell assembly formation in the jumping links and brainnetome 12.5% model. The figure illustrates the effects of varying neural noise on the formation of cell assemblies (CAs) for action- and object-related words in the jumping-links and brainnetome 12.5% model. Background noise was varied from 0, 5, 10, 15 (see Table S2). The results show that changes in neural noise primarily affected overall CA size and activation magnitude, while having comparatively little impact on the topographical distribution differences between action- and object-related word representations.

Appendix A

Structure and function of the spiking model

Each of the 12 simulated areas is implemented as two layers of artificial neuron-like elements ("cells"), 625 excitatory and 625 inhibitory, thus resulting in 15,000 cells in total. Each excitatory cell "*e*" consists of a leaky integrate-and-fire neuron with adaptation and simulates a single pyramidal cell representative of excitatory spiking activity in a cortical microcolumn, while its twin inhibitory cell "*i*" is a graded-response cell simulating the average inhibitory response of the cluster of interneurons situated in a local neighborhood (Eggert and van Hemmen, 2000; Wilson and Cowan, 1972). The state of each cell *x* is uniquely defined by its membrane potential $V(x,t)$, specified by the following equation:

$$\tau \cdot \frac{dV(x,t)}{dt} = -V(x,t) + k_1(V_{In}(x,t) + k_2\eta(x,t)) \qquad (1.1)$$

where $V_{In}(x,t)$ (defined by equation 1.2) is the net input acting upon cell *x* at time *t* (sum of all inhibitory and excitatory postsynaptic potentials – I/EPSPs; inhibitory synapses are given a negative sign), τ is the membrane's time constant, k_1, k_2 are scaling values (see Table 2 for the specific parameter values used in the simulations) and $\eta(\cdot,t)$ is a white noise process with uniform distribution over [-0.5,0.5]. Note that noise is an inherent property of each model cell, intended to mimic the spontaneous activity (baseline firing) of real neurons. Therefore, noise was constantly present in all areas, in equal amounts (inhibitory cells have k_2=0, i.e., the noise is generated by the excitatory cells in the model for convenience).

$$V_{In}(\mathrm{x},\mathrm{t}) = -k_G\omega_{\mathrm{G}}(A_x,t) + \sum_{\forall \mathrm{y}} \mathrm{w}_{\mathrm{x,y}} \cdot \phi(y,t) \qquad (1.2)$$

In Equation (A1.2), *y* varies over all cells in the network, $w_{x,y}$ is the weight of the link from *y* to *x*, and $\phi(y,t)$ is *y*'s current output (1 or 0), as defined below (A2); $\omega_G(A_x,t)$ is the area-specific (or "global") inhibition for area *A* where cell *x* is located (see explanation below and Eq. 3.3): this term is identical for all excitatory cells *x* in *A* and absent for inhibitory cells (k_G as scaling constant).

The weights of inhibitory synapses are assigned a negative sign. The output (or transformation function) ϕ of an excitatory cell *e* is defined as follows:

$$\phi(e,t)\begin{cases}1 & \text{if } (V(e,t)-\alpha\omega(e,t)) > \text{ thresh} \\ 0 & \text{otherwise}\end{cases} \qquad (2)$$

Thus, an excitatory cell *e* spikes (=1) whenever its membrane potential *V*(*e,t*) overcomes a fixed threshold *thresh* by the quantity *αω.*(*e,t*) (where *α* is a constant and *ω* is defined below). Inhibitory cells are graded response neurons as they intend to represent the average impact of a cluster of local interneurons; the output *ϕ*(*i,t*) of an inhibitory neuron *i* is 0 if *V*(*i,t*)<0 and *V*(*i,t*) otherwise.

To simulate neuronal adaptation (Kandel et al., 2012), function *ω*(·,*t*) is defined so as to track the cell's most recent firing rate activity. More precisely, the amount of adaptation *ω*(*e,t*) of cell *e* at time *t* is defined by:

$$\tau_{ADAPT}\cdot\frac{d\omega(e,t)}{dt} = -\omega(e,t) + \alpha_{adapt}\phi(e,t) \qquad (3.1)$$

where τ_{ADAPT} is the "adaptation" time constant. The solution *ω*(*e,t*) of Eq. (3.1) is the low-pass-filtered output ϕ of cell *e*, which provides an estimate of the cell's most recent firing-rate history. A cell's average firing activity is also used to specify the network's Hebbian plasticity rule (see Eq. (A4) below); in this context, the (estimated) instantaneous mean firing rate ω_E(*e*,*t*) of an excitatory neuron *e* is defined as:

$$\tau_{Favg}\cdot\frac{d\omega_E(e,t)}{dt} = -\omega_E(e,t) + \phi(e,t) \qquad (3.2)$$

Local (lateral) inhibitory connections and area-specific inhibition are also implemented, realising, respectively, local and global competition mechanisms (Duncan, 2006; Duncan et al., 1997). More precisely, in Eq. (1.2) the input V_{In}(*x,t*) to each excitatory cell of the same area includes an area-specific ("global") inhibition term $k_G.\omega_G$(*e*,*t*) (with k_G a constant and ω_G(*e*,*t*) defined below) subtracted from the total I/EPSPs postsynaptic potentials V_{In} in input to the cell; this regulatory mechanism ensures that area (and network) activity is maintained within physiological levels (Braitenberg and Schüz, 1998):

$$\tau_{\text{GLOB}}\cdot\frac{d\omega_G(e,t)}{dt} = -\omega_G(e,t) + \sum_{e\in area}\phi(e,t) \qquad (3.3)$$

Excitatory links within and between (possibly non-adjacent) model areas are established at random and limited to a local (topographic) neighbourhood; weights are initialised at random, in the range [0, 0.1]. The probability of a synapse being created between any two cells falls off with their distance (Braitenberg and Schüz, 1998) according to a Gaussian function clipped to 0 outside the chosen neighbourhood (a square of size *n*=19

for excitatory and n=5 for inhibitory cell projections). This produces a sparse, patchy and topographic connectivity, as typically found in the mammalian cortex (Amir et al., 1993; Braitenberg and Schüz, 1998; Douglas and Martin, 2004; Kaas, 1997).

The Hebbian learning mechanism implemented simulates well-documented synaptic plasticity phenomena of long-term potentiation (LTP) and depression (LTD), as implemented by Artola, Bröcher and Singer (Artola et al., 1990; Artola and Singer, 1993). In the model, we discretise the continuous range of possible synaptic efficacy changes into two possible levels, +Δ and − Δ (with Δ<<1 and fixed). Following Artola et al., we defined as "active" any (axonal) projection of excitatory cell e such that the estimated firing rate $\omega_E(e,t)$ of cell e at time t (see Eq. 3.2) is above ϑ_{pre}, where $\vartheta_{\text{pre}} \in [0,1]$ is an arbitrary threshold representing the minimum level of presynaptic activity required for LTP to occur. Thus, given a pre-synaptic cell i making contact onto a post-synaptic cell j, the change $\Delta w(i,j)$ in efficacy of the (excitatory-to-excitatory) link from i to j is defined as follows:

$$\Delta w(i,j) = \begin{cases} +\Delta & \text{if } \omega_E(i,t) \geq \theta_{\text{pre}} \text{ and } V(j,t) \geq \theta_+ (LTP) \\ -\Delta & \text{if } \omega_E(i,t) \geq \theta_{\text{pre}} \text{ and } \theta_- \leq V(j,t) \leq \theta_+ \text{ (homosynaptic } LTD) \\ -\Delta & \text{if } \omega_E(i,t) \leq \theta_{\text{pre}} \text{ and } V(j,t) > \theta_+ \text{ (heterosynaptic LTD)} \\ 0 & \text{otherwise} \end{cases} \quad (4)$$

Table S3. Parameter Values. Model parameter values used in all networks during simulations.

Simulation Parameter Values	
Time constant (excitatory cells)	τ = 2.5 (simulation time-steps)
Time constant (inhibitory cells)	τ = 5 (simulation time-steps)
Total input rescaling factor	k_1 = 0.01
Noise amplitude	$k_2 = 8*\sqrt{(24/\Delta t)}$
Global inhibition strength<br><br>Threshold	 <br>k_G = 120
Spiking threshold	*Thresh* = 0.18
Sensorimotor stimulation amplitude	500
Adaptation strength	α = 7.0
Adaptation time constant	τ_{ADAPT} = 10 (time steps)
Rate-estimate time constant	τ_{Favg} = 15 (time steps)
Global inhibition time constant	τ_{GLOB} = 12 (time steps)
Postsynaptic Membrane Potential Values	
	$\theta+$ = 0.15
	$\theta-$ = 0.14
Presynaptic Output Required for LTP	
	ϑ_{pre} = 0.05
Learning Rate	Δ = 0.008

Reference:

Amir, Y., Harel, M., Malach, R., 1993. Cortical hierarchy reflected in the organization of intrinsic connections in macaque monkey visual cortex. Journal of Comparative Neurology 334, 19–46. https://doi.org/10.1002/cne.903340103

Artola, A., Bröcher, S., Singer, W., 1990. Different voltage-dependent thresholds for inducing long-term depression and long-term potentiation in slices of rat visual cortex. Nature 347, 69–72. https://doi.org/10.1038/347069a0

Artola, A., Singer, W., 1993. Long-term depression of excitatory synaptic transmission and its relationship to long-term potentiation. Trends Neurosci. 16, 480–487. https://doi.org/10.1016/0166-2236(93)90081-v

Braitenberg, V., Schüz, A., 1998. Cortex: Statistics and Geometry of Neuronal Connectivity, 2nd ed. Springer-Verlag, Berlin Heidelberg. https://doi.org/10.1007/978-3-662-03733-1

Douglas, R.J., Martin, K.A.C., 2004. Neuronal Circuits of the Neocortex. Annual Review of Neuroscience 27, 419–451. https://doi.org/10.1146/annurev.neuro.27.070203.144152

Duncan, J., 2006. EPS Mid-Career Award 2004: brain mechanisms of attention. Q J Exp Psychol (Hove) 59, 2–27. https://doi.org/10.1080/17470210500260674

Duncan, J., Humphreys, G., Ward, R., 1997. Competitive brain activity in visual attention. Current Opinion in Neurobiology 7, 255–261. https://doi.org/10.1016/S0959-4388(97)80014-1

Eggert, J., van Hemmen, J.L., 2000. Unifying framework for neuronal assembly dynamics. Phys. Rev. E 61, 1855–1874. https://doi.org/10.1103/PhysRevE.61.1855

Kaas, J.H., 1997. Topographic maps are fundamental to sensory processing. Brain Res. Bull. 44, 107–112. https://doi.org/10.1016/s0361-9230(97)00094-4

Kandel, E.R., Schwartz, J.H., Jessell, T.M., Siegelbaum, S.A., Hudspeth, A.J., 2012. Principles of Neural Science, 5. edition. ed. McGraw-Hill Education Ltd, New York.

Wilson, H.R., Cowan, J.D., 1972. Excitatory and Inhibitory Interactions in Localized Populations of Model Neurons. Biophysical Journal 12, 1–24. https://doi.org/10.1016/S0006-3495(72)86068-5
